# Genomic divergence between two sister *Ostrya* species through linked selection and recombination

**DOI:** 10.1002/ece3.9611

**Published:** 2022-12-15

**Authors:** Jin Zhang, Shangzhe Zhang, Zeyu Zheng, Zhiqiang Lu, Yongzhi Yang

**Affiliations:** ^1^ State Key Laboratory of Grassland Agro‐Ecosystems, College of Ecology Lanzhou University Lanzhou China; ^2^ CAS Key Laboratory of Tropical Forest Ecology, Xishuangbanna Tropical Botanical Garden Chinese Academy of Sciences Mengla China

**Keywords:** gene flow, genomic island, *Ostrya*, resequence, selection

## Abstract

Studying the evolution of genomic divergence between lineages is a topical issue in evolutionary biology. However, the evolutionary forces that shape the heterogeneous divergence of the genomic landscape are still poorly understood. Here, two wind‐pollinated sister‐species (*Ostrya japonica* and *O. chinensis*) are used to explore what these potential forces might be. A total of 40 individuals from 16 populations across their main distribution areas in China were sampled for genome‐wide resequencing. Population demography analyses revealed that these two sister‐species diverged at 3.06–4.43 Mya. Both population contraction and increased gene flow were detected during glacial periods, suggesting secondary contact at those times. All three parameters (*D*_XY_, π, and *ρ*) decreased in those regions showing high levels of differentiation (*F*_ST_). These findings indicate that linked selection and recombination played a key role in the genomic heterogeneous differentiation between the two *Ostrya* species. Genotype–environment association analyses showed that precipitation was the most important ecological factor for speciation. Such environmentally related genes and positive selection genes may have contributed to local adaptation and the maintenance of species boundaries.

## Graphical abstract

Based on the whole genome resequencing data of the two sister *Ostrya* species, we revealed that linked selection and recombination played a key role on the genomic heterogeneous differentiation. Besides, the environment‐related genes and positive selection genes may also contribute to local adaptation and the maintenance of species boundary.

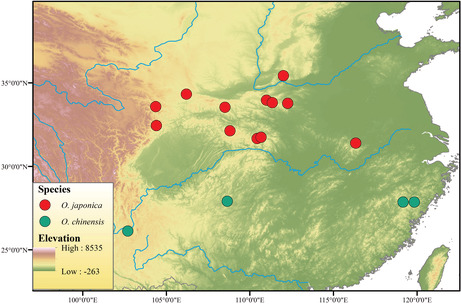

## 1 INTRODUCTION

We gradually gain insight into the evolution from single genes to whole genomes with the development of speciation genomics (Feder et al., 2012). A genome's divergence during speciation always supplies the fundamental data for understanding for how species evolve (Seehausen et al., 2014). Next‐generation sequencing provides a solution to documenting and digging into the landscapes of genomic divergence (Li et al., 2015; Ma et al., 2018; Qiu et al., 2015; Teng et al., 2017; Wang et al., 2018). The divergence across genome is highly heterogeneous and the peaks of differentiation are scattered along whole genome, which have been demonstrated in most genomic speciation studies. These high differentiation regions are generally called “genomic islands,” and its distribution had been explained by many models. However, the evolutionary processes that drive the evolution of genomic islands in different cases remain controversial (Cruickshank & Hahn, 2014; Renaut et al., 2013; White et al., 2010).

Although gene flow is common during the speciation process, it is usually considered as an impediment to speciation (Nosil, 2008). With the gene flow conditions, genomic islands form due to natural selection that resists gene flow, while the rest of the genome shows lower levels of divergence (Nosil et al., 2009; Sousa & Hey, 2013). Only a few loci under strongly divergent selection existed in the early stage of species divergence, which is often considered as reproductive isolation loci (Seehausen et al., 2014), and which then expand in size and number with the help of “divergence hitch‐hiking” to form large differentiated regions (Feder & Nosil, 2010; Seehausen et al., 2014). Thus, in divergent parapatric ecotype populations, their differentiated regions are limited to a few large genomic regions (Andrew & Rieseberg, 2013; Turner et al., 2005). In some cases, however, genomic islands are also thought to form by a sorting of ancient divergent haplotypes (Cruickshank & Hahn, 2014). A high level of genome heterogeneity can be due to the accumulation of variation in coalescent times, while undergoing strong selection accelerates differentiation at non‐neutral loci and reduces shared ancestral variations (Cruickshank & Hahn, 2014). The shaping of landscape of genomic heterogeneous divergence is also determined by recombination (Nachman & Payseur, 2012; Noor & Bennett, 2009). Genomic islands occur more often in low recombination regions (Burri et al., 2015; Ellegren et al., 2012; Wang et al., 2016). In these regions, genetic linkage is stronger, so linked selection will affect larger genomic regions by reducing diversity (Burri et al., 2015). Then, genetic drift will accelerate due to smaller effective population size of reduced diversity, and so promote divergent differences among populations and species (Burri et al., 2015). The landscapes formed under natural conditions are very complicated to disentangle as these models are not mutually exclusive.

The identification of genomic islands and their role in speciation processes have recently become major focuses of speciation research. Studies have involved an extensive range of organisms including insects (Turner et al., 2005), fishes (Jones et al., 2012), plants (Ma et al., 2018), mammals (Zhou et al., 2018), and birds (Han et al., 2017). Wright's fixation index (*F*_ST_; Hudson et al., 1992) is often used to measure divergence, although it may be affected by reduced genetic diversity (Cruickshank & Hahn, 2014). Another parameter that is usually considered to be an absolute measurement of divergence, *D*_XY_, is less affected by the reduction of genetic diversity and can be used in combination with *F*_ST_ to disentangle two factors from neutral processes: divergent selection and gene flow (Seehausen et al., 2014). The elevated *F*_ST_, *D*_XY_ in regions is thought to be elevated due to resistance to gene flow or existence of ancient polymorphisms (Han et al., 2017; Wolf & Ellegren, 2017). When evolutionary process involving recurrent selective sweeps and recent ecological selection, ongoing background selection or linked selection, drive genomic island, *D*_XY_ is expected to decrease or remain unchanged (Han et al., 2017).

*O. japonica* and *O. chinensis* (= *O. multinervis*) are two sister‐species of hop‐hornbeam in the genus *Ostrya* Scop (Turner, 2014). *O. japonica* is widely distributed in the north Asia (China, Japan, and Korea) and in China it covered the north regions, which from the eastern to northwest. On the contrary, *O. chinensis* is an endemic species in China and only contained sparely records in southern and southwestern regions (Lu et al., 2016). Both species are monoecious, primarily outcrossing, deciduous, and rarely reproduce clonally (Huang et al., 2017). These two closely related species are geographically distinct and morphologically differentiated. Thus, to assess how evolutionary forces shape genomic landscapes during speciation, *O. japonica* and *O. chinensis* are valuable research materials. Here, we performed the sequencing of whole genome across their biogeographical distribution in China to reveal their genomic differentiation patterns during their divergence. By analyzing their phylogenetic and population structure, we aimed to separate our sampled individuals into two groups that we hypothesized should coincide with the two species. Both species underwent population contraction during glaciations when gene flow also increased. This suggests the possibility of secondary contact between the two species. We also aimed to identify differentiated genomic islands by quantifying *F*_ST_ and testing whether natural selection, linked selection, recombination, or gene flow were the major driving forces of island formation. Moreover, we also explore the positive selection of genes related to environmental adaptation, since these may not only have adapted them to local conditions but may also have accelerated and maintained their differentiation into two species.

## 2 MATERIALS AND METHOD

### 2.1 Sample collection, sequencing, and mapping

**FIGURE 1:**
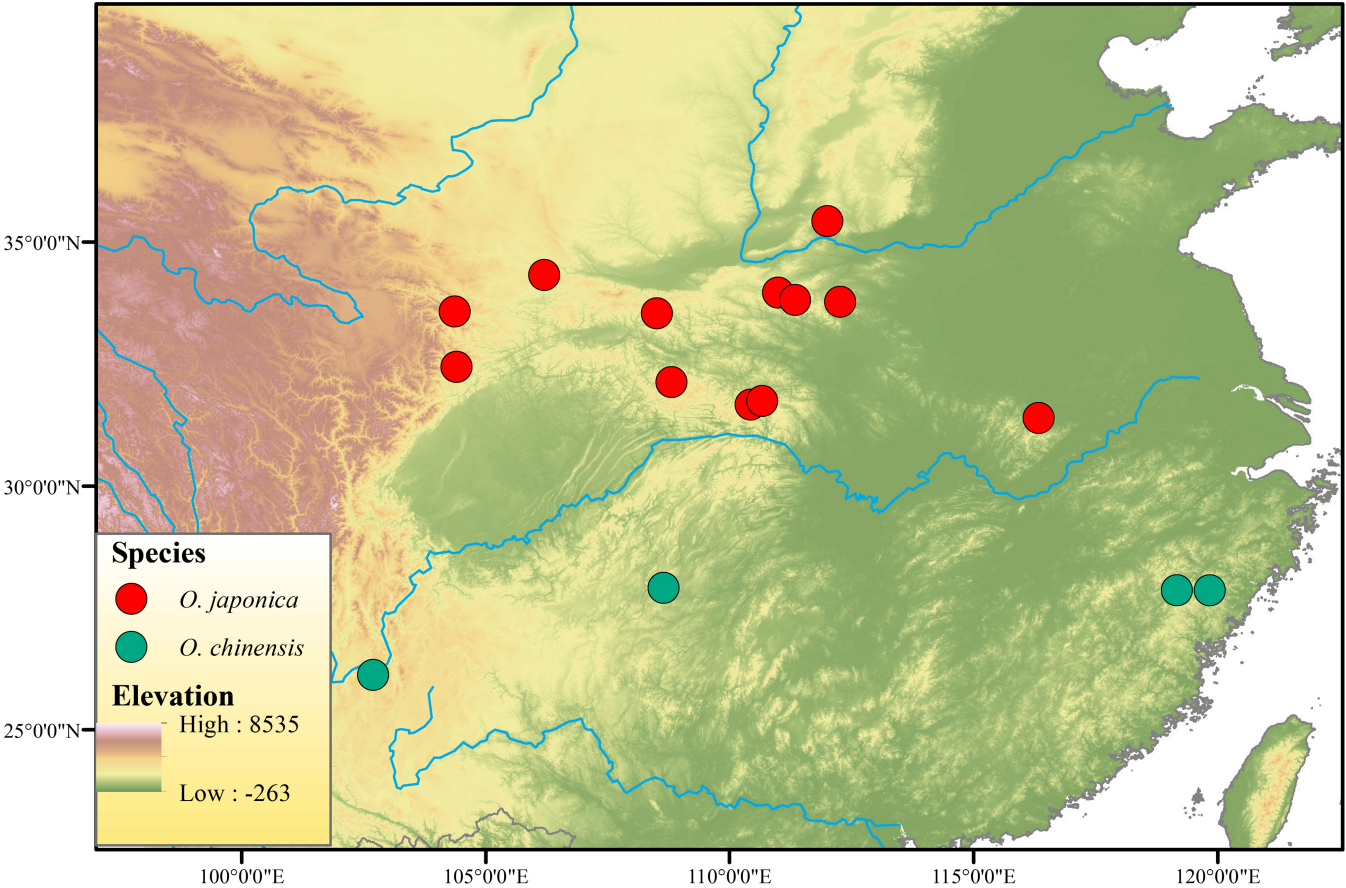
The geographic map of sequenced *O. japonica* and *O. chinensis* individuals. Orange circles represent *O. japonica* and green circles represent *O. chinensis*.

We first examined the distribution of *O. japonica* in China based on the CVH database (https://www.cvh.ac.cn/). To reduce the repeat samples of each population, only two trees at least 50 m apart, we collected healthy fresh leaves and dried them using silica gel. A total of 26 *O. japonica* trees from 12 populations across its geographic distribution in China (Figure 1) were sampled for genome‐wide resequencing (Table S1). The CTAB method (Del Sal et al., 1989) was performed to extract whole genomic DNA and then sequenced using the Illumina Hi‐seq 2000 platform. A total of 14 *O. chinensis* samples and two *Carpinus cordata* individuals were obtained from our previous study (NCBI BioProject: PRJNA428015; Yang et al., 2018). We then used fastp (Chen et al., 2018) v 0.23.2 to filter the low‐quality sequences with the following parameters: “‐M 20 ‐5 ‐3 –l 50.” We used BWA‐MEM method v 0.7.12 (Li, 2013) to map the clean reads to *O. rehderiana* reference genome with default parameters. SAMtools v 1.10 (Danecek et al., 2021) was then selected sorted the aligned results and remove any duplicated reads. We used Genome Analysis Toolkit (GATK, v 3.7; McKenna et al., 2010) to mark the putative regions around InDels and performed the realignment analyses around those regions to improve mapping quality.

### 2.2 SNP and genotype calling

We converted the BAM files to the GVCF format files using HaplotypeCaller method from GATK; then called the genotype using the GenotypeGVCFs method. InDels and SNPs were selected by “Selectvariants” module within GATK, and then the GATK officially recommended filter parameters of “HardFilter” were performed on these variants as follow: “QD < 2.0; FS > 60.0; QUAL < 30.0; MQ < 40.0; MQRankSum < ‐12.5; ReadPosRankSum < ‐8.0” for SNPs, and “QD < 2.0; FS > 200.0; QUAL < 30.0; ReadPosRankSum < ‐20.0” for InDels. To get the high‐quality SNP sites, we further filtered the results under the following conditions: (i) we deleted the SNP sites that locate around the 5 bp region of InDels; (ii) we marked SNPs as missing if their coverage was lower than one‐third of or higher than three time the average depth and removed the SNP sites with a missing rate >20%, and (iii) we removed the SNP sites in repeat regions.

### 2.3 Phylogenetic relationships analyses

*Carpinus cordata* and *C. fangiana* individuals were also collected (NCBI BioProject: PRJNA428015) as outgroups and mapped to the *O. rehderiana* genome using the same method as described above. We used concatenated data sets of SNPs to generate a neighbor‐joining tree. We calculated a pairwise genetic distance matrix and generated the neighbor‐joining tree with the “dnadist” and “neighbor” programs in PHYLIP v 3.69.660 (Felsenstein, 1993). In addition, we extracted the single‐copy genes from each individual which satisfied the following two criteria: a gene coverage rate >90% and a gene average depth should within 0.5 to 1.5 times of the average depth of the individual. A total of 9678 single‐copy genes were found among all samples; these were used to perform the maximum‐likelihood tree inferred by IQ‐TREE v 2.1.2 (Nguyen et al., 2015). Finally, ASTRAL v 5.7.8 (Mirarab et al., 2014) was used to infer the species tress with gene trees.

### 2.4 Population structure and identity by descent analysis

We executed a principal component analysis (PCA) using “smartpca” program within EIGENSOFT v 6.1.4 (Price et al., 2006) and used the Tracy–Widom test to show the degree of clustering. Population structure was generated based on all SNP sites by ADMIXTURE v 1.3 (Alexander et al., 2009). Admixture proportions were calculated with the quasi‐Newton convergence acceleration algorithm. We considered the hypothetical population number *K* value to be from 1 to 5; with the flag “‐‐cv” every *K* value state can be cross‐validated. The *K* value whose cross‐validation error was lowest considered to be the best result. We then used BEAGLE v 4.0 (Browning & Browning, 2013) to perform the identity by descent (IBD) analysis with the parameters: “window = 10000; overlap = 1000; ibd = true; ibdtrim = 100.” This allowed the identification of shared blocks between each pair of individuals.

### 2.5 Demographic history inference and ecological niche modeling analyses

First, we performed a Multiple Sequentially Markovian Coalescent (MSMC) model (Schiffels & Durbin, 2014) to investigate the demographic history of *O. japonica* and *O. chinensis*. Four individuals from each population were selected, and SNPs were generated with SAMtools and BCFtools v 1.10 (Danecek et al., 2021) according to the guidelines of MSMC. We then set 10 years as generation time and set 2.182 × 10^−8^ per locus per generation as mutation rate (Yang et al., 2018), and they were used to infer effective population size. We also estimated a folded joint site frequency spectrum for the two species with ANGSD v 0.933 (Nielsen et al., 2012), and then used fastsimcoal2 software (Excoffier et al., 2013) to investigate the history of population separation between *O. japonica* and *O. chinensis*. A total of fourteen models were considered, each performed 100 times as independent bootstrapping simulations. The best‐fitting model was selected as that for which the Akaike Information Criterion estimate was the smallest. That model was then used to calculate parameter intervals from a run of 100 simulations.

In order to deeply understand the historical distribution of the two species, we performed the ecological niche modeling analyses by MAXENT (Phillips & Dudík, 2008). We collected the distribution points of *O. japonica* and *O. chinensis* in China (Table S5) and download the 19 environmental factors (or called bioclimatic variables) of last glacial maximum (LGM) and recent (1970–2000) from WorldClim for these region. These data were filtered by removing highly correlated (|*r*| > .8) environmental factors using ENMTools (Warren et al., 2021) and a total of 8 factors (bio2, bio3, bio5, bio6, bio7, bio15, bio18, bio19) were retained. Then SDMtoolbox (Brown, 2014) was used all to reduce the occurrence localities. Finally, MAXENT was ran with the following parameters: random test of 25%, background points of 10,000, replicates of 10, and maximum iterations of 1000.

### 2.6 Inferring population parameters

The nucleotide diversity (*π*) and pairwise genetic divergence(*F*_ST_) were calculated by VCFtools v 0.1.13 (Danecek et al., 2011) within and between two species, respectively. The recombination rates along each chromosome (*ρ* = 4Ner) were calculated in population scale by FastEPRR (Gao et al., 2016). Mean pairwise nucleotide difference (*D*_XY_) between the two species was calculated according to the formula (Cruickshank & Hahn, 2014):

$$D_{\mathrm{XY}}=\sum_{\mathrm{ij}}x_{i}y_{j}d_{\mathrm{ij}}$$

where *x*_*i*_ represents the *i*th sequences in species *X* and *y*_*j*_ represents the *j*th sequences in species *Y*. The total nucleotide differences of the two sequence (*i*th and *j*th) were denoted as *d*_*ij*_. All parameters were estimated with the 20‐kb window and 5‐kb step‐size; windows including fewer than 10 SNPs were discarded.

### 2.7 Identifying the divergent regions

Divergent regions were identified by the following procedures. To avoid the influence of random factors, we first discarded windows with the SNP number less than 10. Windows with the highest *F*_ST_ (top 1%) across the whole genome were selected and then combined by permutation and empirical approaches (Feulner et al., 2015; Ma et al., 2018) to determine outlier windows. For each window, the same amount of SNPs were permuted from the whole‐genome SNPs and estimated these *F*_ST_ value. A total of 5,000,000 permutations were performed for each window to generate the null *F*_ST_ distributions. We then estimated the *p*‐values of experimental windows using null *F*_ST_ distributions, and all the *p*‐values were corrected by FDR method. We defined the outlier windows belong to experimental windows whose FDR lower than 0.01. Finally, we combined the adjacent outlier windows to form the final divergent regions.

### 2.8 Copy number variation (CNV) analyses

The read depth‐based software Control‐FREEC (Boeva et al., 2011) was used to identify the CNV regions for each sample. The default program parameters were used except for “coefficientOfVariation = 0.062; window = 10000; step = 2000.” Genes containing CNV regions were defined as CN genes (CNGs). The number of copy numbers overlapping with CNGs was counted, and the average copy number of each gene was designated as the gene's copy number.

The outlier CNGs between *O. japonica* and *O. chinensis* were also inferred based on the parameter (*V*_ST_) described by David et al. and given by the formula in (Rinker et al., 2019):

$$V_{\mathrm{st}}=\frac{V_{\text{total}}-\left(V_{\mathrm{Oja}}\times N_{\mathrm{Oja}}+V_{\mathrm{Och}}\times N_{\mathrm{Och}}\right)/N_{\text{total}}}{V_{\text{total}}}$$

where *V*_total_, *V*_Oja_, and *V*_Och_ are the copy number variance for total individuals, *O. japonica* population, and *O. chinensis* population, respectively; *N*_total_, *N*_oja_, and *N*_och_ are the sample sizes of total individuals, the *O. japonica*, and the *O. chinensis* populations, respectively. *V*_ST_ which fell in the top 5% of all genes were defined as outliers. To further estimate the intraspecific CN homogeneity and interspecific differentiation for all CNGs, we used a permutation method. We randomly permutated all individuals into two cluster according to the individuals of the two species and generate a new *V*_ST_ for each outlier, and then 1000 iterations were constructed null distribution of *V*_ST_. Finally, the *p*‐value for each CNG outlier was estimated and just only these outliers whose *p*‐value ≤.01 were defined as CN differentiation genes (CNDGs).

### 2.9 Positively selected genes analyses

We used the classic Hudson–Kreitman–Aguadé test (HKA; Hudson et al., 1987) to identify the positively selected genes (PSGs). In brief, the *F*_ST_ of each gene was calculated; we counted the number of SNPs within one population (= N_1_) and the number of fixed loci (*F*_ST_ ≥ 0.95) between two populations (= N_2_). For each gene, we performed 2 × 2 contingency table filled with N_1_(gene), N_2_(gene), and the genome‐wide average N_1_(genome) and N_2_(genome). The null hypothesis was N_1_(gene):N_2_(gene) = N_1_(genome):N_2_(genome), and we used Pearson's chi‐square test to compare the ratio of N_1_(gene):N_2_(gene) to N_1_(genome):N_2_(genome). Only genes whose a *p*‐value ≤.01 from the HKA, and more than one nonsynonymous site which annotated and predicted by SnpEff (Cingolani et al., 2012), were considered as PSGs for each group.

### 2.10 Genotype–environment associations analysis

We downloaded climate and weather data (19 bioclimatic variables, logged every 30 s, ~1 km^2^) from WorldClim (https://www.worldclim.org) and downloaded the soil database from FAO (https://www.fao.org). We collected the relevant environmental data for each individual and calculated correlation coefficients for each environmental predictor using the function *pairs.panels* in R (Figure S13). After removing environmental autocorrelation (|*r*| ≥ .7), we derived seven environmental predictors as follows: subsoil sand fraction (SSF), subsoil pH (Sp), solar radiation (SR), max temperature (MaT), min temperature (MiT), mean diurnal range (MDR), and annual precipitation (AP; Figure S14). We used PLINK v1.90 (Purcell et al., 2007) for SNPs pruning based on LD with 0.4 *r* ^2^ threshold, and set 20‐kb window with two makers per step. Using these criteria, 0.28 M SNPs were selected. Redundancy analysis (RDA; Forester et al., 2018) was used to explore the relationship between environments and variations of SNPs and CNGs. We chose significant constrained axes (*p* < .01) and determined those outlier variations loaded in the tails of distributions (three standard deviations) in these constrained axes. Finally, we used the *cor*, one R function to calculate the correlations of each outlier's variations with the seven environmental predictors.

To evaluate the false positives that may due to neutral variations in genotype‐environment associations analysis, we performed the following analyses. A total of 23,622 SNPs located in intergenic regions, which were considered neutral variations. Then the Isolation‐by‐distance analyses and Isolation‐by‐environment analyses (Sang et al., 2022) were performed between environment‐associated SNPs and neutral SNPs. The *F*_ST_/(1 − *F*_ST_) between natural populations was calculated for environment‐associated SNPs and neutral SNPs, respectively. The Mantel method in *vegan* was used to test for associations between *F*_ST_/(1 − *F*_ST_) and geographic and environmental distance.

## 3 RESULTS

### 3.1 Genome resequencing and population structure

In total, 268‐Gb sequencing data were generated for the 26 *O. japonica* and 14 *O. chinensis* sampled across China. The average depth was 21.28× and an average genome coverage of 90% was detected for each individual (Table S1). After variant calling and subsequent filtering, we identified high‐quality SNPs about 3.6 Mb. Among them, 0.38 Mb SNPs were shared by both species, which amounted to 17.54% and 27.47% of the total SNPs in *O. japonica* and *O. chinensis*, respectively (Figure S1).

**FIGURE 2:**
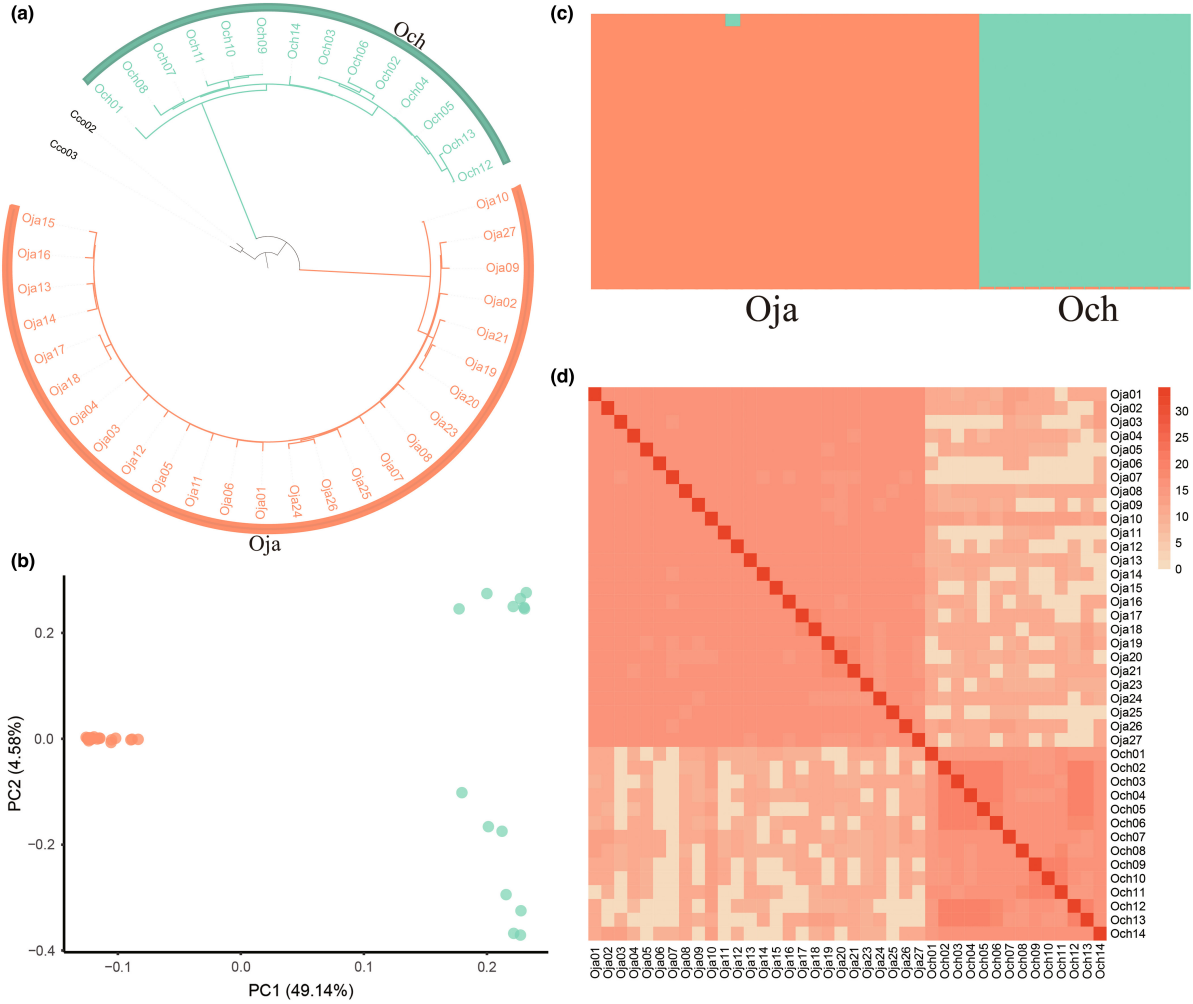
Population structure analyses. (a) A neighbor‐joining (NJ) phylogenetic tree based on SNPs from whole‐genome resequencing data. (b) The first two principal components of PCA showing the distribution of all individuals. (c) Lineage relationships showed by population genetic structure with *K* = 2. (d) Identity‐by‐descent (IBD) analysis showing shared haplotype of all individuals. Colors in Heat‐map indicate the length of IBD blocks (log(bp)). The three‐letter abbreviations represent Oja for *O. japonica* and Och for *O. chinensis*.

To clarify the evolutionary relationships of all samples, we executed a phylogenetic analysis using pairwise genetic distances. Two individuals of *C. cordata* were selected as the outgroup and the neighbor‐joining tree showed that each species clustered distinctly (Figure 2a). We also extracted the single‐copy genes from each individual and created a gene tree, which was similar to the SNP tree (Figure S2). The PCA results (Figure 2b) corroborated the finding since the two species were clearly separated on the first component (PC1, explained 49.14%, *p* = 2.28 × 10^−3^; Table S2). Population structure analyses inferred by ADMIXTURE (Alexander et al., 2009) further indicated there to be a significant lineage differentiation between the two species within the best fitness *K* = 2 (Figures 2c and S3). Only one individual of *O. japonica* exhibited a small amount of components from the *O. chinensis* lineage. The IBD analysis also demonstrated that a large amount of IBD blocks were shared by these two species (Figure 2d), indicating extensive introgressions in their history or the common inheritance of many ancestor haplotypes.

### 3.2 Demographic history

**FIGURE 3:**
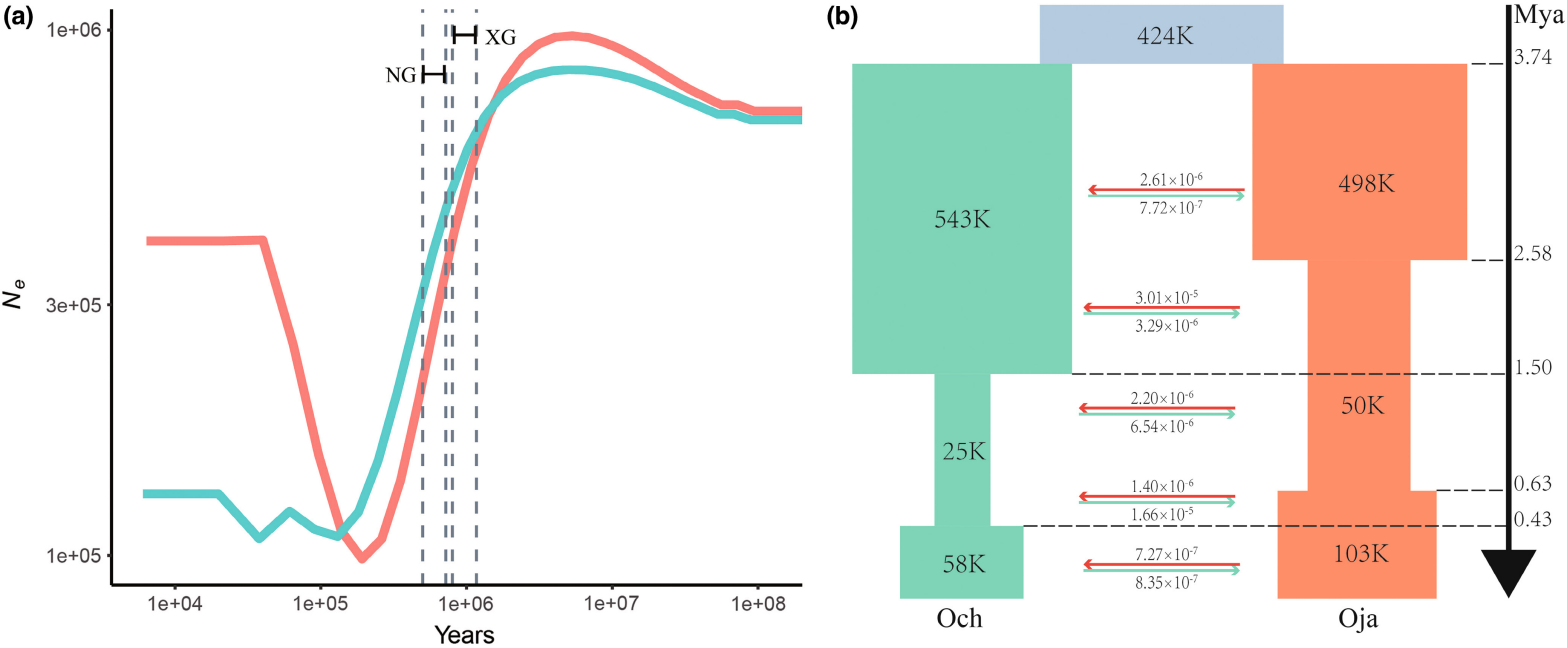
Demographic histories of *O. japonica* (Oja) and *O. chinensis* (Och). (a) Population history result from MSMC model. The curve represents the change in effective population size (*Ne*) from present to ancient. The dashed interval perpendicular to the year axis represents the glacial period, including XG (the Xixiabangma Glaciation, 1.17–0.8 Mya) and NG (the Naynayxyngla Glaciation, 0.72–0.5 Mya). (b) Schematic diagram of the best model results inferred by fastsimcoal2 (Table S4; Figure S5). Estimates of gene flow (horizontal arrow) between lineages are the migration fraction per generation. The red color represents *O. japonica* and the green color represents *O. chinensis*.

The effective change in population size (*Ne*) of each species as revealed by the MSMC model showed both species to have a similar maximum *Ne* size at ~2.0 Mya. This was followed by a dramatic decline in *Ne* to almost one tenth, during the development of two major glaciations: the Xixiabangma Glaciation (XG, 1.17–0.8 Mya) and Naynayxyngla Glaciation (NG, 0.72–0.5 Mya; Zheng et al., 2002; Figure 3a). *O. japonica* then underwent a population expansion after ~0.2 Mya recovering its *Ne* to nearly half its previous maximum value, while *O. chinensis* showed only a little population recovery after ~0.1 Mya. Using Fastsimcoal2 (Excoffier et al., 2013) analyses with the joint folded spectrum, we simulated a total of 14 models of historical divergence of the two species, including isolation, individual migration, group expansion, and/or group bottlenecks (Figure S4 and Table S3). A speciation model in which the divergence of the two species was accompanied by gene flow and involved one population bottleneck and one population expansion per species, gave the highest fit (Figures 3b, S5 and S6; Table S4). The best‐supported model confirmed that *O. japonica* and *O. chinensis* probably diverged at ~3.74 Mya (95% highest posterior density = 3.06–4.43 Mya). Both species then experienced two effective population size change events. *O. japonica* and *O. chinensis* experienced the first bottlenecks during 2.58–0.63 and 1.50–0.43 Mya, respectively, each followed by population expansion, which findings are generally consistent with the MSMC results. We also detected a continuous asymmetric bidirectional gene flow during their divergence, in which there was a stronger gene flow from *O. japonica* to *O. chinensis* at the early diverging stage, and in reverse a stronger gene flow from *O. chinensis* to *O. japonica* occurred in the more recent period. We also detected extensive stronger gene flow between 2.58 and 0.43 Mya than was seen either in the early divergence stage (3.74–2.58 Mya) or during the more recent period (<0.43 Mya). We further investigated the potential distribution regions of these two species by the ecological niche estimation. We found that *O. japonica* migrated to the south which was warmer than the north during the LGM. The distributions of *O. japonica* and *O. chinensis* were highly overlapping, while when the climate warms up after glacial, *O. japonica* expanded to the north and *O. chinensi* continued to expand in south (Figure S7).

### 3.3 Genomic divergence between two species

**FIGURE 4:**
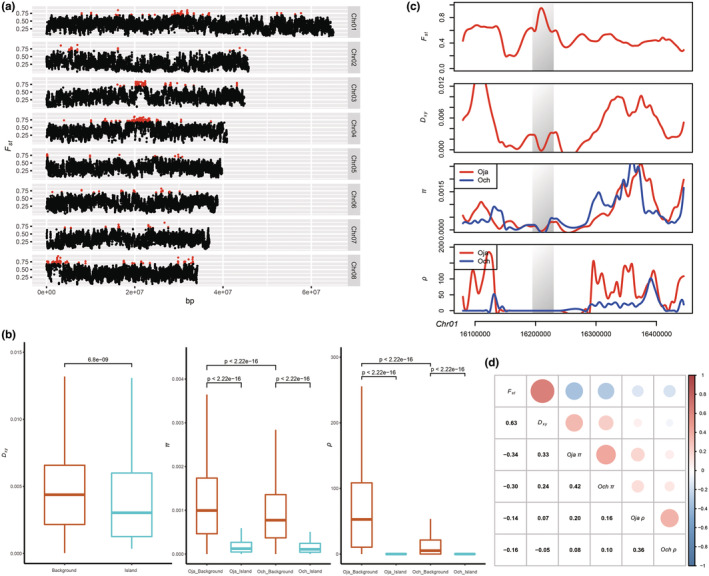
The genomic landscape of divergence between *O. japonica* (Oja) and *O. chinensis* (Och). (a) The distribution of *F*_*ST*_ values across the whole genome (20‐kb windows with 5‐kb step‐size). Genomic islands are shown in red. (b) Wilcoxon test of *D*_XY_, π, and *ρ* between genome‐wide background (orange) and genomic islands (green). (c) Distribution of population genomic parameters along an example chromosome segment (Chr01 16,070,001–16,455,000 bp), with gray bar area referring to a genomic island (Chr01 16,195,001–16,230,000 bp). (d) Correlation of population genomic parameters (*F*_ST_, *D*_XY_, π, and *ρ*). Red and blue represent positive correlation and negative correlation, respectively. The circle size and color bias refer to the absolute value of Spearman's correlation coefficient.

The genome‐wide population characters were evaluated by the sliding 20‐kb window with a 5‐kb step‐size (Table S6). *O. japonica* showed a higher nucleotide diversity (*π*; 1.26 × 10^−3^ ± 8.31 × 10^−6^, 95% HPD) than observed in *O. chinensis* (1.03 × 10^−3^ ± 7.51 × 10^−6^, T test *p* < 2.2 × 10^−16^). This may be due to the *O. japonica Ne* being larger than that of *O. chinensis* in recent time (Figure 3b). The average genetic divergence (*F*_ST_) was found to be 0.347, and the mean absolute genetic divergence (*D*_XY_) was 4.59 × 10^−3^ ± 2.34 × 10^−5^. The *F*_ST_ values showed a U‐shaped distribution with 461,371 fixed divergence sites accounting for 12.81% of the total number of SNPs in the two species (Figure S8), and which could be due to reduced introgression in recent time. We also found, in common with previous studies, the genomic divergence to be highly heterogeneous, regardless of whether the level of divergence is high or low (Figure 4a; Brawand et al., 2014; Burri et al., 2015; Ma et al., 2018). The parameters *F*_ST_, *D*_XY_, *π*, and *ρ* underwent further statistical tests of correlation. *F*_ST_, although obviously highly positively correlated with *D*_XY_ (*r* = .63, *p* < 2.2 × 10^−16^), was negatively correlated with both *π* and *ρ* (Figure 4d). High positive correlation coefficients were also detected between *π* and *D*_XY_ and between *π* and *ρ*, while a low correlation coefficient was identified between *D*_XY_ and *ρ* (Figure 4d).

To learn more about evolutionary forces shaping the genomic divergent landscape, we first identified outlier windows based on their *F*_ST_ values. We identified a total of 607 outlier windows with 20‐kb size (Figure 4a; Table S7) according to a previously described method (Feulner et al., 2015; Ma et al., 2018). These outlier windows were not distributed at random throughout the genome. However, they were significantly located in chromosomes 3, 4, and 8 (chi‐square test, *p* < .01, Table S7), where they exhibited different distributions being concentrated in the middle of chromosomes 3 and 4, and at the head of chromosome 8 (Figure 4a). After examining their genomic distribution and combining adjacent outlier windows, we obtained the final genomic islands, most of them being small and containing only one window (Figure S9).

Nucleotide diversity was also examined. Comparing with the genome background, the *π* values of these islands were significantly reduced strongly (Figure 4b,c). We further compared the *D*_XY_ values for genome background and genomic islands in order to examine which factors might contribute to genomic islands' formation (Cruickshank & Hahn, 2014; Han et al., 2017). A significantly reduced *D*_XY_ value appeared when recovered from genome islands (Figure 4b,c). This finding differs from the results of studies where the adopted scenario is of speciation with recent gene flow or where islands have been derived from divergent sorting of ancient divergent haplotypes (Cruickshank & Hahn, 2014; Han et al., 2017). The recombination rate (*ρ*) analyses showed that the *ρ* value for islands was significantly lower compared to the *ρ* value for genome background (Figure 4b,c). In addition, since *ρ* = 4*Ne*r, where *r* is the per‐generation recombination rate, a reduction of *Ne* in the regions linked to selection affects the estimation of *ρ*, which will be underestimated even if *r* is similar to other regions in the genome. We also performed a further comparison of *ρ*/*π* that could obtain a measure of the recombination rate independent of local *Ne* (Ellegren et al., 2012; Wang et al., 2016) from which the results showed that *ρ*/*π* was still significantly lower in the islands (Figure S10). This observation, combined with the lower *D*_XY_ value, may indicate recurrent background selection mainly form genomic islands (Han et al., 2017; Wang et al., 2016).

### 3.4 Positively selected genes and copy number variation analyses

Using a HKA test (Hudson et al., 1987), 2821 PSGs were identified. Their functions were mainly involved in DNA repair, metabolism, and flower development (Table S8). A total of 105 PSGs were located in genomic islands (Table S9), which also showed a significantly biased distribution within islands (*p* < 2.2 × 10^−16^). These were associated with their response to UV, DNA repair, and the cell surface receptor signaling pathway (Table S10). Among such genes, many perform important functions in plant development (Table S9). For example, *GCR1* showed an major role in seed germination and flowering, the overexpression of *GCR1* in *Arabidopsis* will reduce the seed dormancy and the time to bloom (Colucci et al., 2002). *ABCB1* appears through mediating polar auxin transport to regulate photomorphogenesis and root development in *Arabidopsis* (Lin & Wang, 2005). *UVR8* is one important UV‐responding genes, which could orchestrate the expression of protective genes, and it also involved in controlling aspects of leaf growth and morphogenesis (Gruber et al., 2010; Wargent et al., 2009).

We further estimated the divergence between the two species by whole‐genome CNVs. We identified 3220–4454 CNVs across a number of different individuals, among which there was a total of 16,918 CN genes (CNGs, that contain CNV regions). By permuting all individuals 1000 times and calculating *V*_ST_, a total of 846 CNDGs were identified with significant copy number differentiated differences between the two species (*p* < .01, top 5% *V*_ST_; Figure S11). These genes were mainly involved in secondary metabolism, response to light, and iron ion transport (Table S11). Moreover, a total of 37 CNDGs were distributed in genomic islands, which is a significant enrichment when compared to only 252 genes located in islands (*p* < 2.2 × 10^−16^, Figure S12).

### 3.5 Environment adaptation‐related variations

**FIGURE 5:**
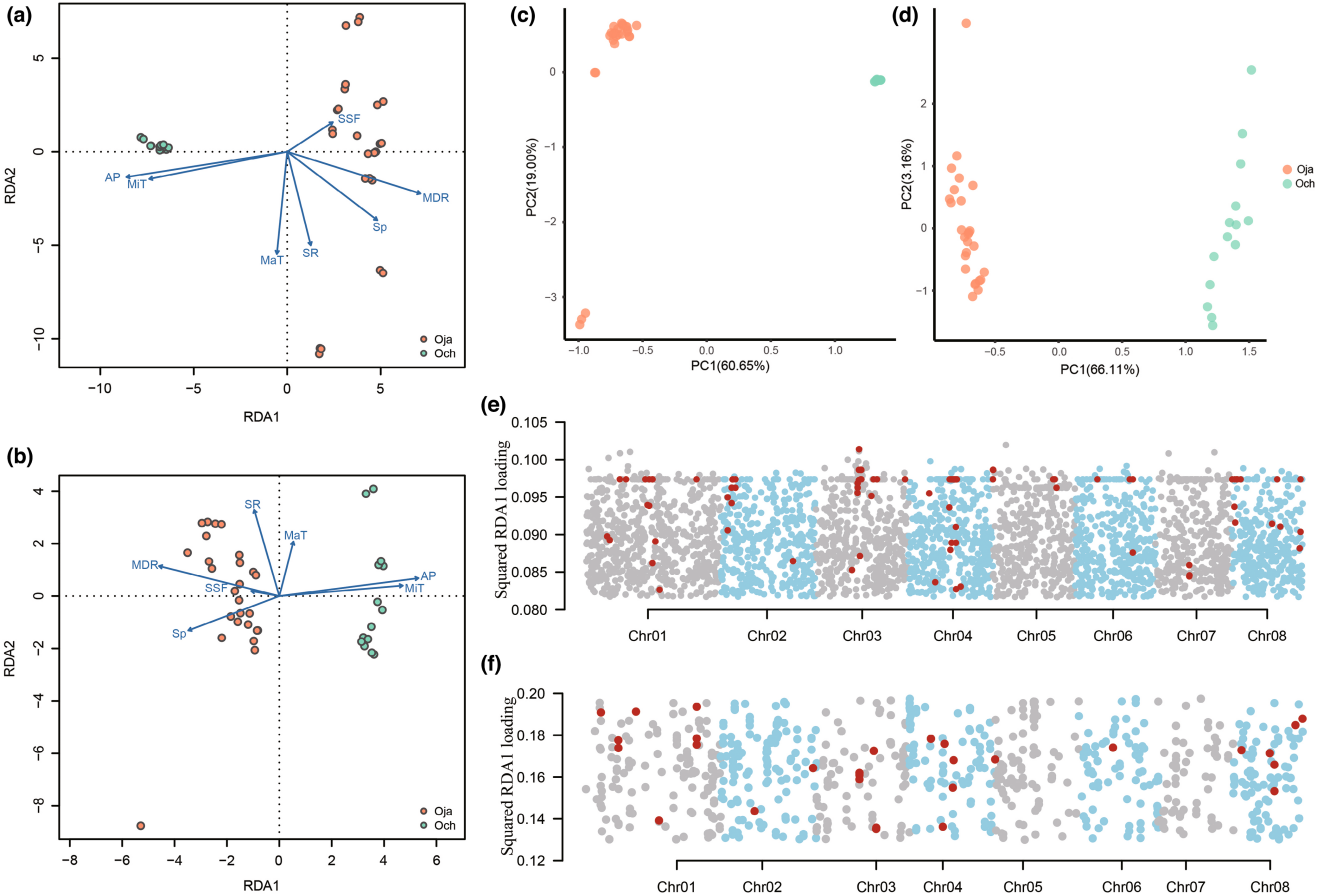
The RDA of *O. japonica* (Oja) and *O. chinensis* (Och). (a) and (b) represent the relationships between the independent environmental parameters and population structure based on SNPs and CNGs, respectively. The shadow length of the environmental variables onto each constrained axis is proportional to the influence of the individual's distribution on that axis. (c) and (d) represent the PCA results based on outlier variations (SNPs and CNGs). (e) and (f) represent the distributions of outlier SNPs and CNGs on the first constrained axes (RDA1). The red points represent variations inside the genomic islands.

We further used the RDA approach to explore the candidate variations (SNPs and CNGs) between the two species, which may relate to the environmental adaptation. Seven environmental predictors with low correlations were selected (Figures S13 and S14), including SSF, Sp, SR, MaT, MiT, MDR, and AP. Through the RDA analyses, we found all the predictors explained 10.48% and 10.12% of SNPs and CNGs variances, respectively (Table S12), and only the first one or two constrained axes (RDA1‐2) are significantly (*p* < .01) correlated with these genomic variances (Table S12). Both RDA1 axes were found to be the most important vectors (SNPs explained 3.37%; CNGs explained 4.37% of the variance) dividing the two species into two clusters (Figure 5a,b). Both sets of results showed that *O. japonica* prefers environments in which MDR and Sp are higher, and where Ap and MiT are lower than that preferred by *O. chinensis*. This finding is consistent with the significant differences detected among these same predictors within the two species (Table S13). We also identified the outlier genomic variations as defined by a 3× standard deviation (two‐tailed *p* = .0027) cutoff from the mean along each significantly constrained axis. A total 4825 outlier SNPs and 990 outlier CNGs were identified, both of which distinguish the two species by PCA analyses (Figure 5c,d), indicating the two species to be significantly adapted to different environments. Using the Pearson correlation threshold of |*r*| > .6 between the environmental factors, we found annual precipitation to be the most relevant correlate for both types (Figure S15), suggesting that water stress may be the main constraint on their distribution. A total 1958 and 990 genes were related to the outlier SNPs and outlier CNGs, respectively; their functions were mainly related to basic physiological activities, stress resistance, and reproduction (Tables S14 and S15).

We also investigated the distribution of outlier genomic variations in relation to environmental adaptation. The outlier SNPs and CNGs appeared to be randomly distributed along each chromosome, few of them being located in islands (Figures 5e,f and S16). Nearly one‐third of all outlier SNP‐associated genes overlapped with the PSGs, indicating a significant level of enrichment (319 genes, chi‐square test, *p* < .01). We also found the RDA1outlier SNPs, which contain the major outliers, showed a much higher *F*_ST_ value than the background, while the RDA2 outliers had no such increase of *F*_ST_ (Figure S17). A total of 23,622 neutral SNPs (located in the intergenic regions) were further identified and used to calculate *F*_ST_/(1 − *F*_ST_) between natural populations with outlier SNPs and neutral SNPs. The outlier SNPs displayed significant association with geographic and environmental distance, but the neutral SNPs not (Figure S18). This suggests that outlier SNPs are more closely related to environmental/geographic heterogeneity, and also suggests that neutral variations may have little impact on our results. Furthermore, only 38 outlier CNGs located in genomic islands (Figure 5f) and there were 745 outlier CNGs as CNDGs (Figure S11), indicating environmental factors also mainly drive CNV differentiation.

## 4 DISCUSSION

Here, we have characterized the evolutionary histories of two sister forest tree species with a distinct distribution between North and South China by population genomic analyses. The results highlight how a variety of evolutionary process effected divergence of genome‐wide patterns. Our population structure and demography analyses showed that *O. japonica* and *O. chinensis* both formed a clear and independent lineage and they diverged around 3.74 Mya during the middle Pliocene. Both species exhibited population contraction during the glacial period (~1 Mya) and showed a high level of gene flow around 1.5 Mya, suggesting the secondary contact may exist during such geological events and accelerate the gene flows (Komaki et al., 2015; Liu et al., 2014; Figure 3). This results further supported the similarity of the distribution locations of *O. japonica* and *O. chinensis* during LGM, indicates that the two species may have the same glacial refugiums (Figure S7). With the low rate of recent gene flow and the modern‐day geographic isolation (Figure 3b), the two species have a mean *F*_ST_ value of 0.347 with a U‐shaped distribution more characteristic of allopatric speciation.

Genome islands are affected by many factors that vary among different species. Gene flow is one such factor; when species diverge with recent gene flow, their genomic islands will cluster into just a few larger, discrete genomic islands (Andrew & Rieseberg, 2013; Turner et al., 2005). Under such gene flow model, both *F*_ST_ and *D*_XY_ in genomic islands are expected to increase because of restricted gene flow (Cruickshank & Hahn, 2014). However, we found the size of these islands to be small, with an asymmetric distribution (Figure 4a), and a much lower *D*_XY_ value than the genome background (Figure 4b). This provides some evidence to reject the speciation‐with‐gene‐flow scenario in favor of the selection‐in‐allopatry model without recent gene flow (Cruickshank & Hahn, 2014; Irwin et al., 2016; Nachman & Payseur, 2012). Moreover, this *D*_XY_ distribution pattern also could reject the model of islands formed by divergent sorting of ancient divergent haplotypes (Han et al., 2017; Ma et al., 2018). Our results, in which *F*_ST_ and the population recombination rate (*ρ*) were significantly negatively correlated (Figure 4d) and islands exhibited significantly lower *ρ* values and *ρ*/*π* values (Figures 4b and S10), are in accord with natural selection being the dominant driver of genetic differentiation leading to the *F*_ST_ value increasing in regions where recombination is low (Burri et al., 2015; Ellegren et al., 2012; Wang et al., 2016). Linked selection, where populations' common ancestor might be more recent, means a low value of *π* (Cruickshank & Hahn, 2014; Irwin et al., 2016), which is consistent with our finding lower *π* values in islands than in the genome background (Figure 4b). Thus, such conditions are likely to suit the two sister *Ostrya* species, where natural selection, recurrent linked selection, and recombination played a key role in differentiating their heterogeneous genomic landscape.

There were also obvious differences in the adaptations of *O. japonica* and *O. chinensis* to local environmental conditions. Although both the PSGs and CNDGs showed a significant overlapping with the identified islands, the overlap number was small suggesting the selection of non‐island regions may important during their divergence (Hu et al., 2022). The functions of PSGs and CNDGs were mainly related to plant development, responses to stimuli, and reproduction (Tables S8 and S11), which, being fundamental processes in a plant's life‐cycle, may have been responsible for the reproductive isolation of the two species. Moreover, the RDA‐identified genes that contained the environmentally associated variants (SNPs and CNGs) also showed a significant degree of overlap with PSGs and CNDGs (Figure S11), and the environmentally associated SNPs also exhibited increased *F*_ST_ values (Figure S17). Both these factors have important roles in driving species differentiation. Among the environmental predictors, MDR, Sp, AP, and MiT all had significant impact on the divergence for the two species, but AP was the most significant factor and being related to water is vital to the survival of plants. Various plant organs develop in response to water availability, such as leaves (transpiration) and roots (absorption of water). Our RDA analyses detected multiple related genes, about 10 genes related to leaf developments (e.g., *TAF10*, *UVR8*, and *MEX1*), and about 28 genes related to roots (e.g., *XEG113*, *SINAT5*, and *PIP2‐2*, Table S16). Among them, *TAF10* is abundantly expressed in vascular tissue; its overexpression in *Arabidopsis* causes deformed leaves (Furumoto et al., 2005). *MEX1* was differentially expressed in mutant trichomes (Jakoby et al., 2008). *O. japonica* and *O. chinensis* have distinct differences in their leaf phenotypes regarding venation and trichomes, suggesting that the development of these phenotypes may be regulated by these genes. We also detected 12 genes responding to light, for example, *AOC*, *CRY1*, and *HD16* (Table S16), even though the correlation between SR and variation was low, indicating that the genes may not be affected by a single factor. Such gene variations between the two species also need gene functional analysis to be undertaken in the future so as to reveal clearly how molecular differences affect their phenotypes.

In conclusion, our results have revealed the evolutionary scenario of the two sister *Ostrya* species to be a selection‐in‐allopatry model without recent gene flow between them. Rather than gene flow and sorting of ancient polymorphisms, we found natural selection, recurrent linked selection, and recombination to have taken a key role in the development of their genomic heterogeneous differentiation landscape. The results from PSGs, CNDGs, and RDA provided additional evidence that selection accelerated the divergence of the two species and may have shaped their phenotypic differences, although further functional experiments need to be conducted in future studies to verify this.

## AUTHOR CONTRIBUTIONS

**Jin Zhang:** Conceptualization (equal); data curation (equal); formal analysis (equal); investigation (equal); methodology (equal); validation (equal); visualization (equal); writing – original draft (equal). **Shangzhe Zhang:** Data curation (equal); formal analysis (equal); methodology (equal). **Zeyu Zheng:** Formal analysis (equal); methodology (supporting). **Zhiqiang Lu:** Conceptualization (equal); funding acquisition (equal); investigation (equal); methodology (equal); resources (equal); writing – review and editing (equal). **Yongzhi Yang:** Conceptualization (equal); funding acquisition (equal); investigation (supporting); methodology (supporting); project administration (equal); resources (equal); writing – original draft (supporting); writing – review and editing (supporting).

## CONFLICT OF INTEREST

The authors state that there is no conflict of interest.

## FUNDING INFORMATION

This work was supported by the National Natural Science Foundation of China (Grant No. 31900201) and Ph.D. Programs Foundation of Department of Education of Gansu (Grand No. 2021QB‐007).

### OPEN RESEARCH BADGES

This article has earned Open Data and Open Materials badges. Data and materials are available at [[insert provided URL(s) on the Open Research Disclosure Form]].

## Supporting information


Appendix S1

## Data Availability

The raw sequencing data of *Ostrya japonica* had been submitted into the NCBI database under the BioProject No. PRJNA872315. For *O. chinensis* and *Carpinus cordata*, we used our previously published sequence data (NCBI: PRJNA428015). The scripts and the fastsimcal2 running models used in this study are available at: https://github.com/ZJin2021/SCRIPT_ostrya.
