# Supplementary material for: Genomic divergence between two sister Ostrya species through linked selection and recombination
Source: Ecol Evol. 2022 Dec 15;12(12):e9611. doi: 10.1002/ece3.9611 (PMC9754895; doi:10.1002/ece3.9611)
Supplement: Supplementary file 1 — Appendix S1 [file ECE3-12-e9611-s001.docx]

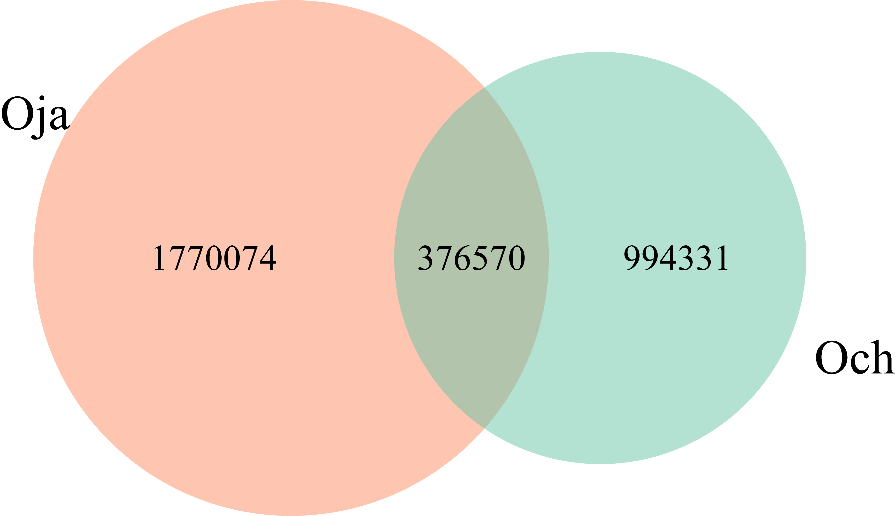


**Figure S1. Unique and shared SNPs between the *O. japonica* (Oja) and *O. chinensis* (Och).**


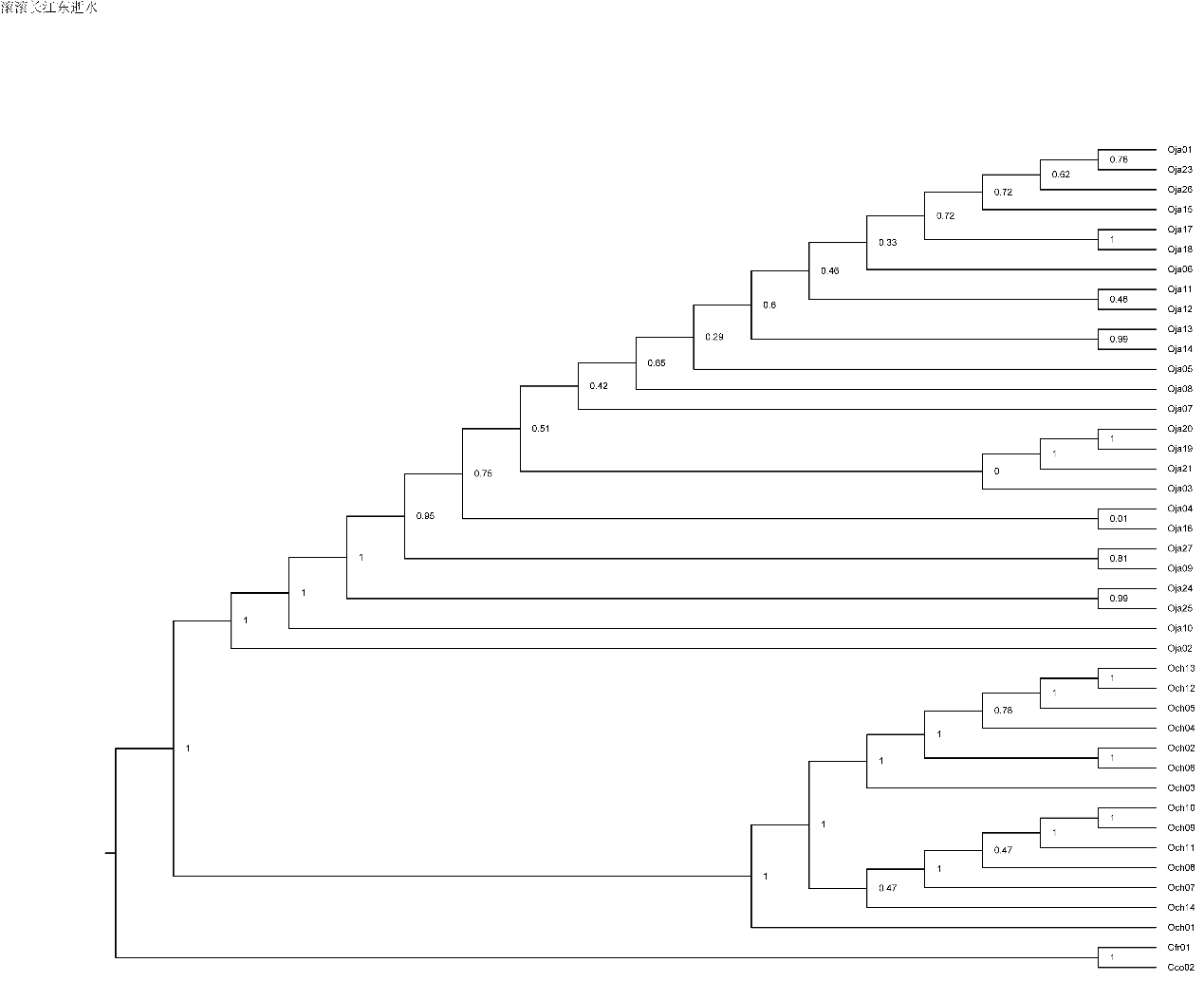


**Figure S2. The coalescent-based maximum-likelihood tree among all individuals of** ***O. japonica* (Oja) and *O. chinensis* (Och).**


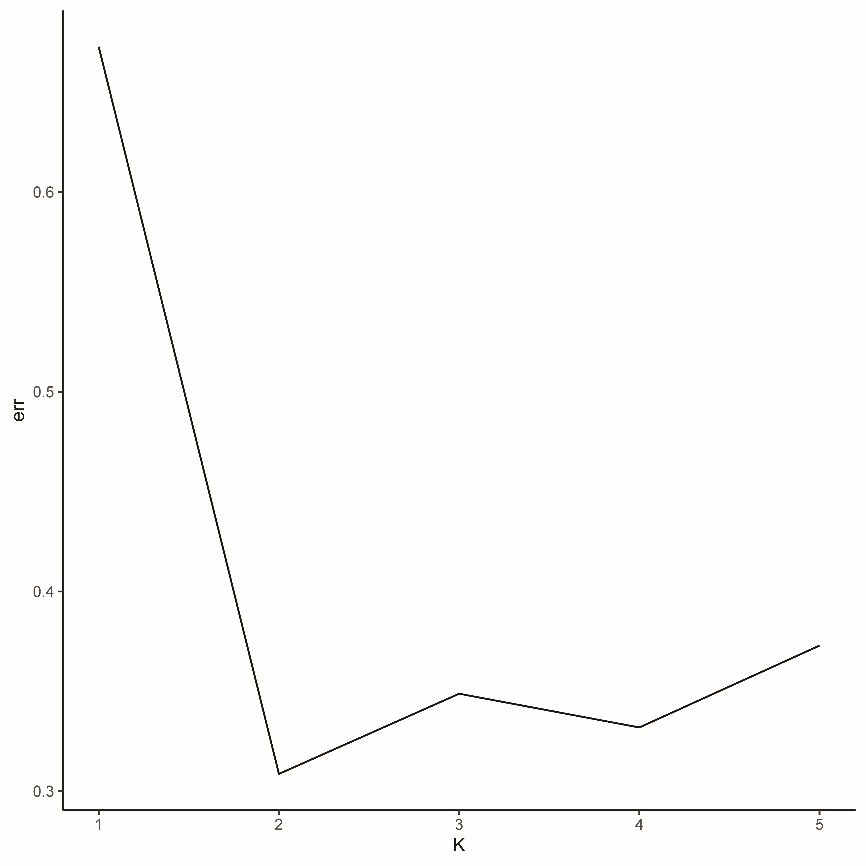


**Figure S3. cross validation results from K=1 to K=5.**


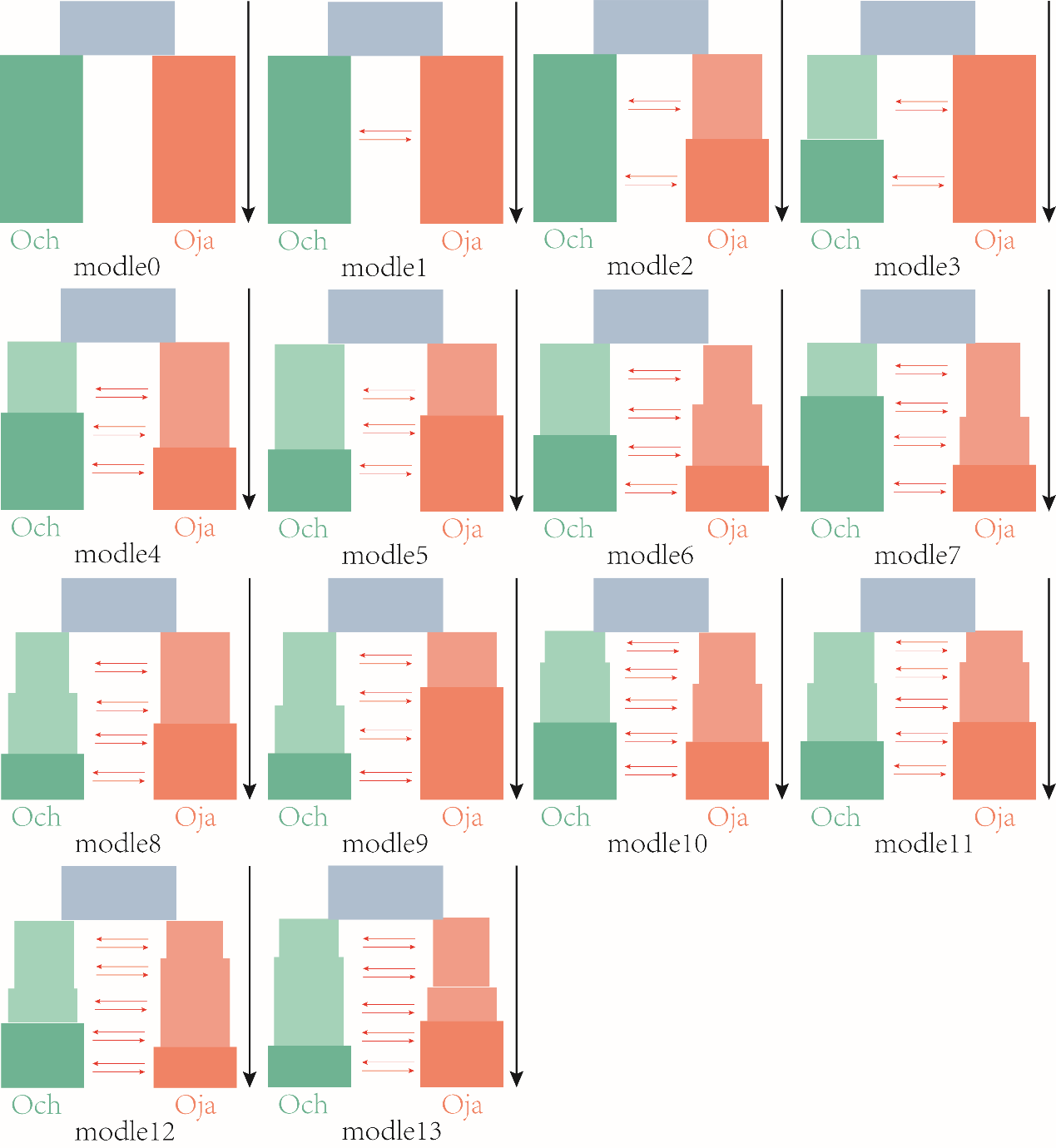


**Figure S4 Schematic diagram of possible topological structures used in fastsimcoal2 to infer demographic parameters.** Red arrows represent gene flow, black arrows represent divergence time, and changes in the width of the rectangles represent changes in effective population size.


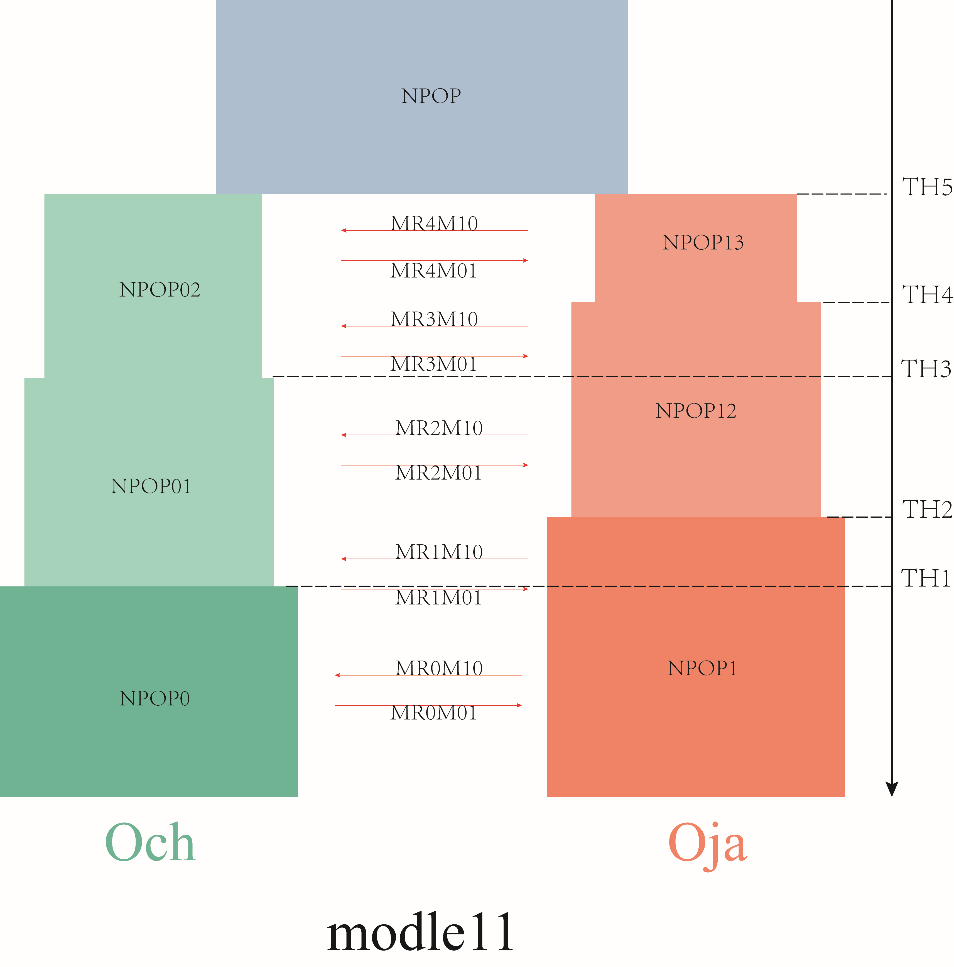


**Figure S5. Schematic diagram of optimal model parameters in fastsimcoal2.** The demographic histories of *O. japonica* (Oja) and *O. chinensis* (Och) were showed. The parameter inside the rectangle represents the effective population size. TH1-TH5 represent the event times. The direction of the horizontal arrow represents the direction of gene flow, and the next parameter represents the value of gene flow.

**
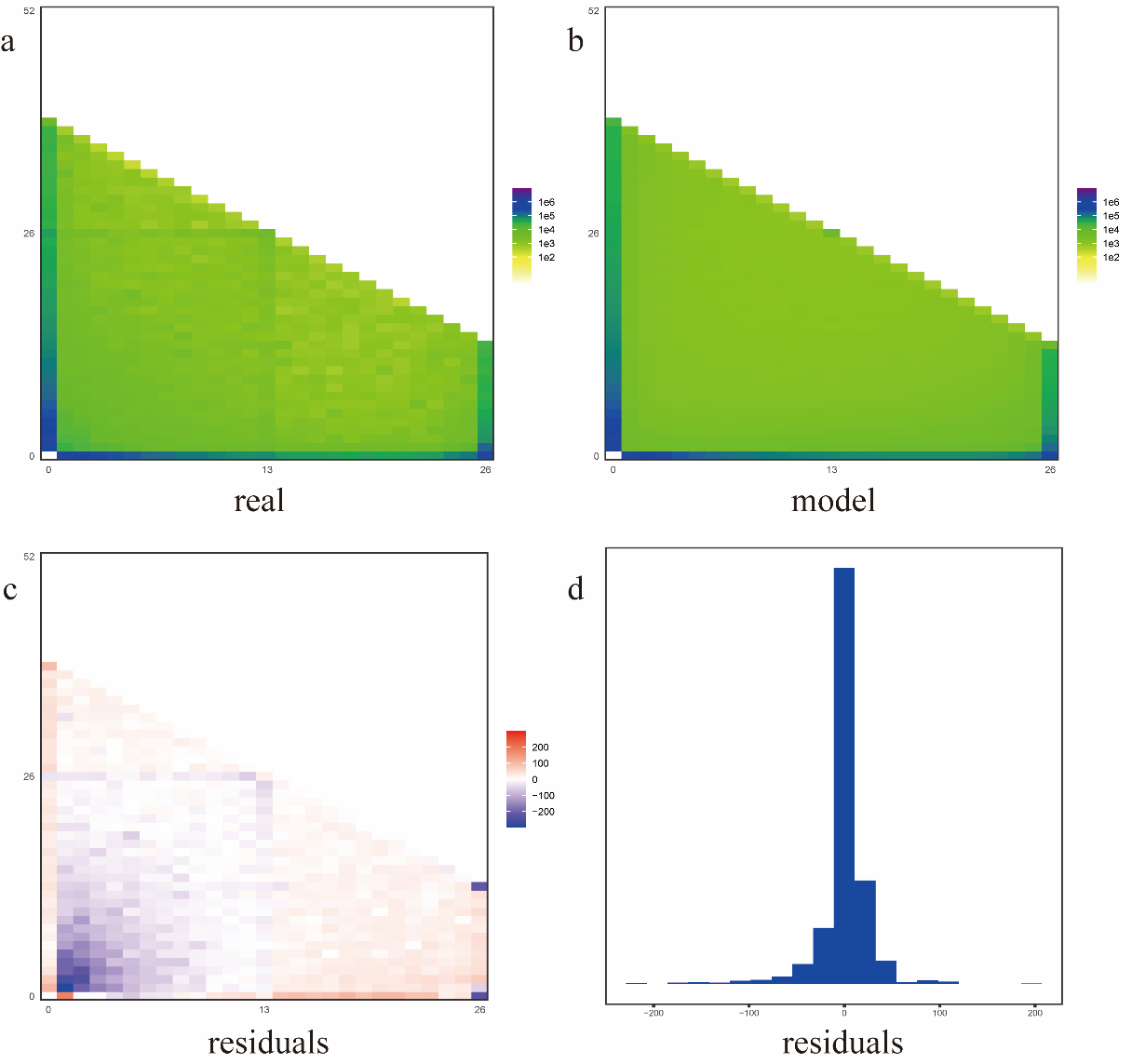
**

**Figure S6. Comparison between observed SFS (real) to the predicted SFS (model11).**

**
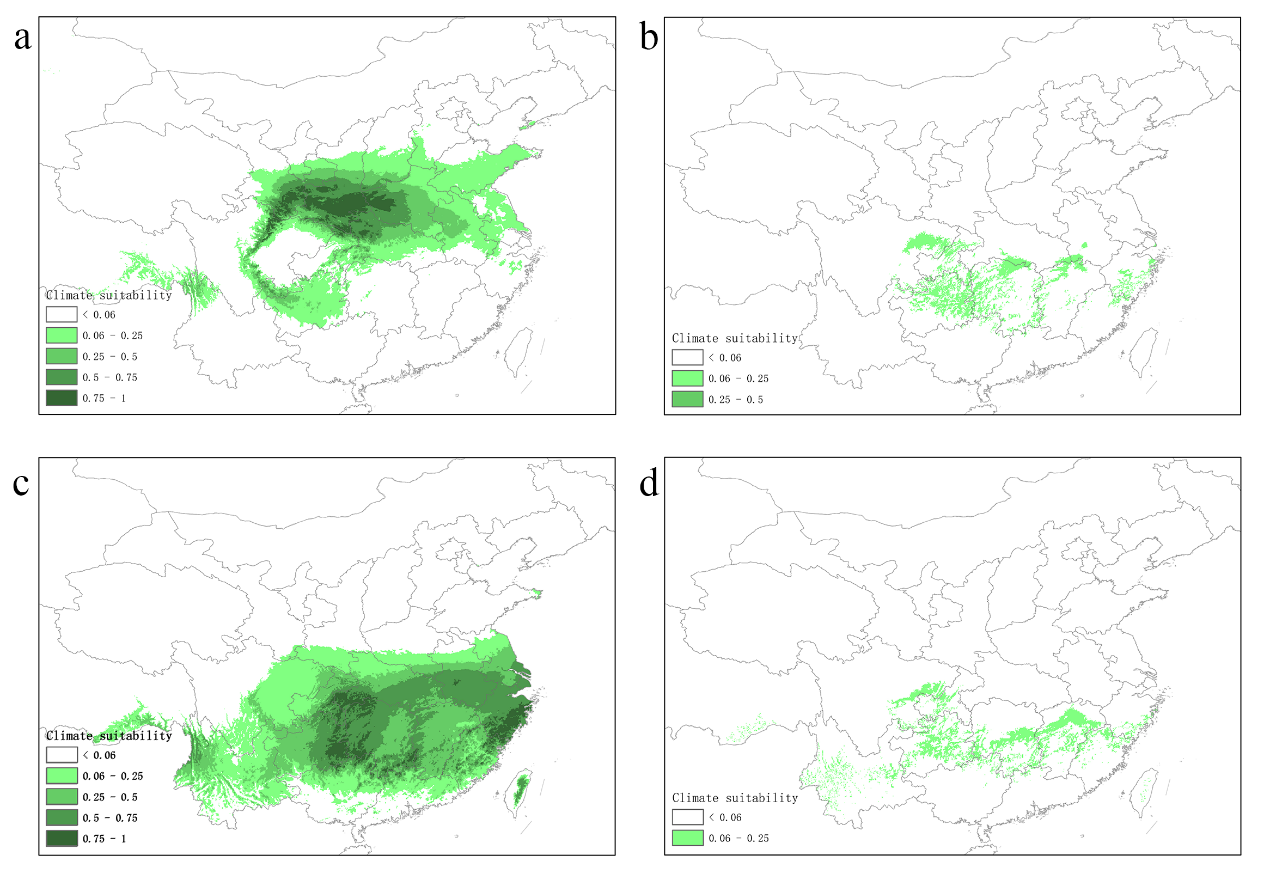
**

**Figure S7. Distribution prediction using MAXENT.** (a) and (c) The current potential distributions of *O. japonica* and *O. chinensis*, the higher the Climate Suitability, the more likely the species is to grow in the area. (b) and (d) The potential distributions of *O. japonica* and *O. chinensis* during the Last glacial maximum (LGM), the higher the Climate Suitability, the more likely the species is to grow in the area.


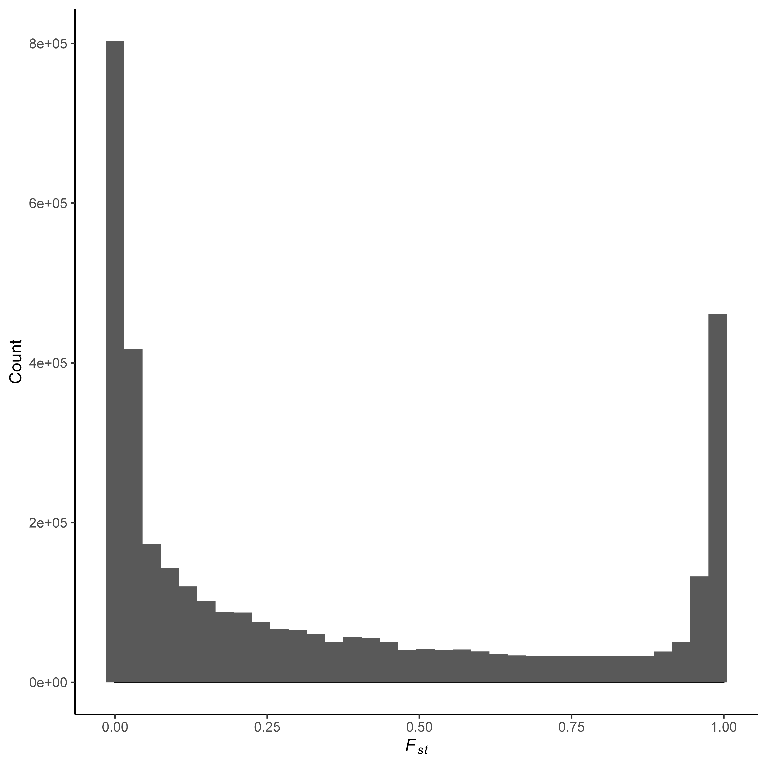


**Figure S8.** ***F*_ST_ distribution along whole-genome**


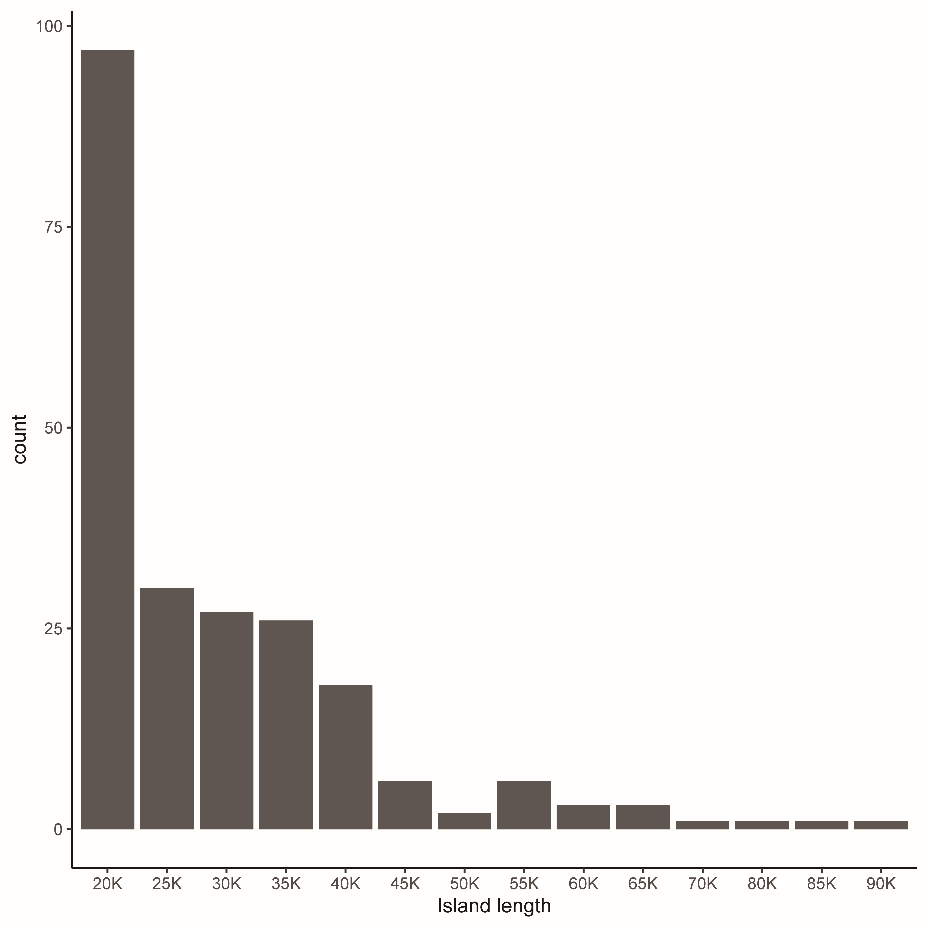


**Figure S9. The size distributions of genomic islands between *O. japonica* and *O. chinensis*.**

*
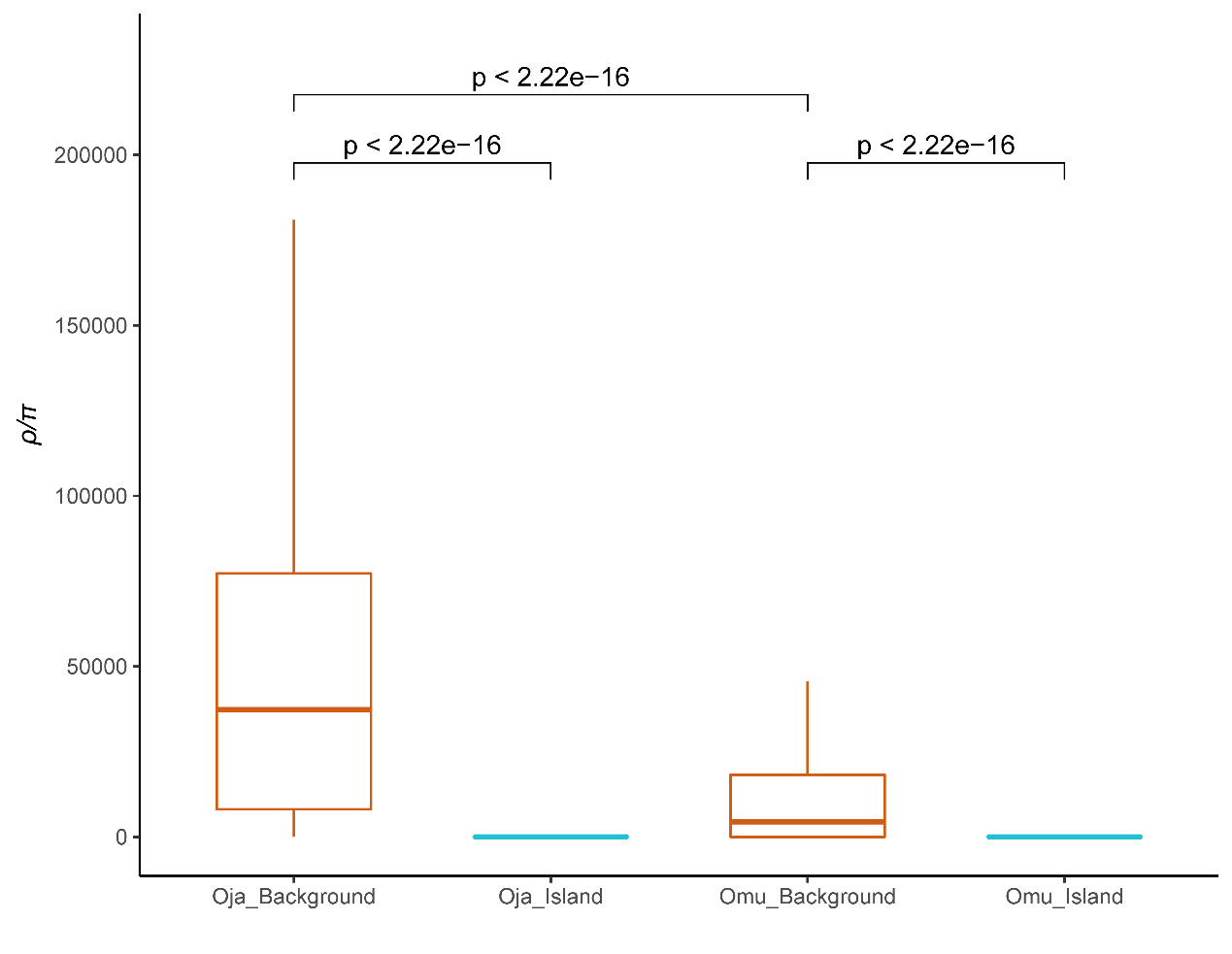
*

**Figure S10. Comparison of population recombination rate (*ρ*) / nucleotide diversity (*π*) between whole-genome (Background) and genomic islands for *O. japonica* (Oja) and *O. chinensis* (Och).** The Wilcoxon tests were performed. The orange represents the genome-wide windows and the blue represents the windows of the island.


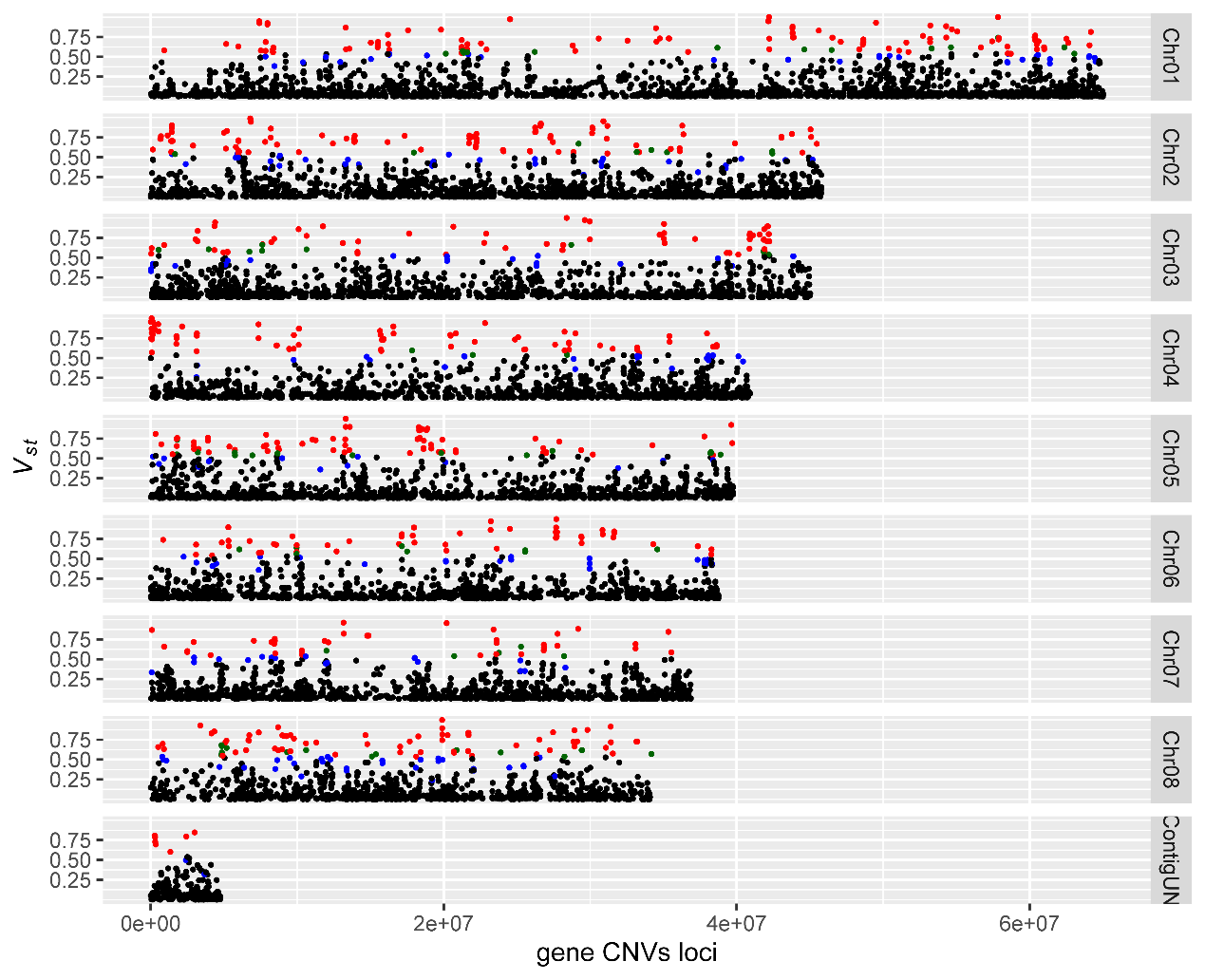


**Figure S11. The distribution of *V*_ST_ values of CN genes (CNGs) on chromosomes.** The red and green points showed the CN differentiation genes (CNDGs) with significant and high difference between *O. japonica* and *O. chinensis*, and the red and blue points showed the outlier CNGs defined by RDA .


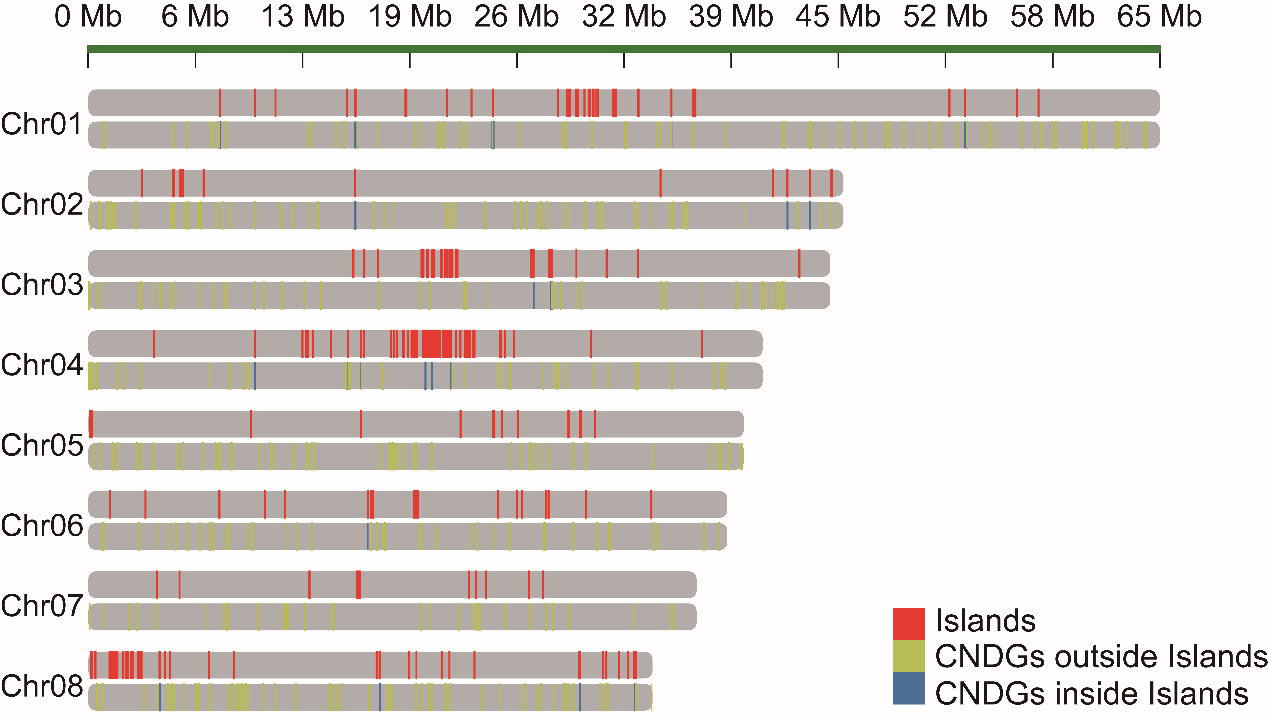


**Figure S12. The distribution of gene islands (top) and CNDGs (bottom) on chromosomes.** Red areas refer to genetic islands, and blue and yellow areas represent CNDGS inside or outside the island, respectively.


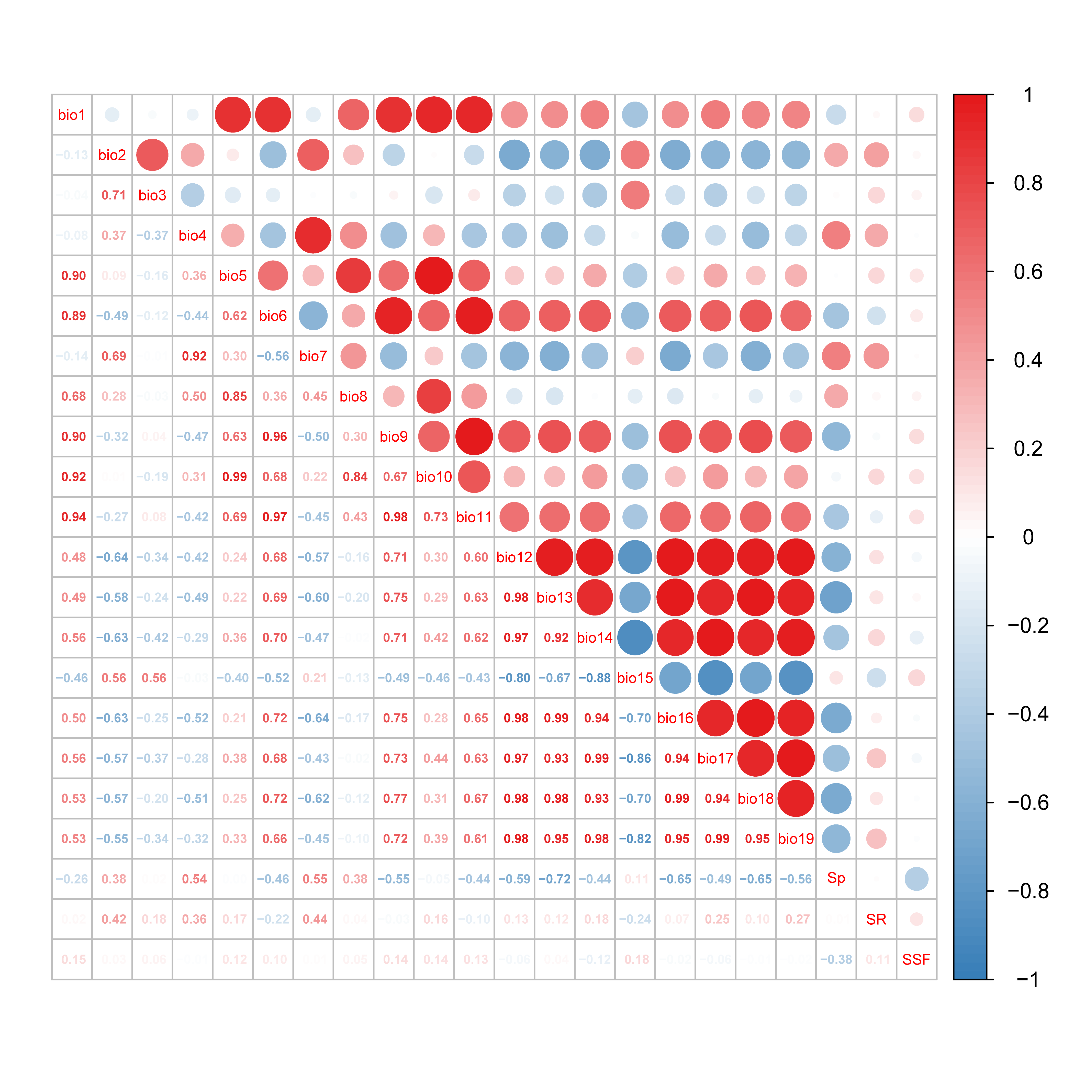


**Figure S13. Correlations between unfiltered environmental predictors. Red represents positive correlation and blue represents negative correlation.** The size of the circle is positively correlated with the correlation coefficient.


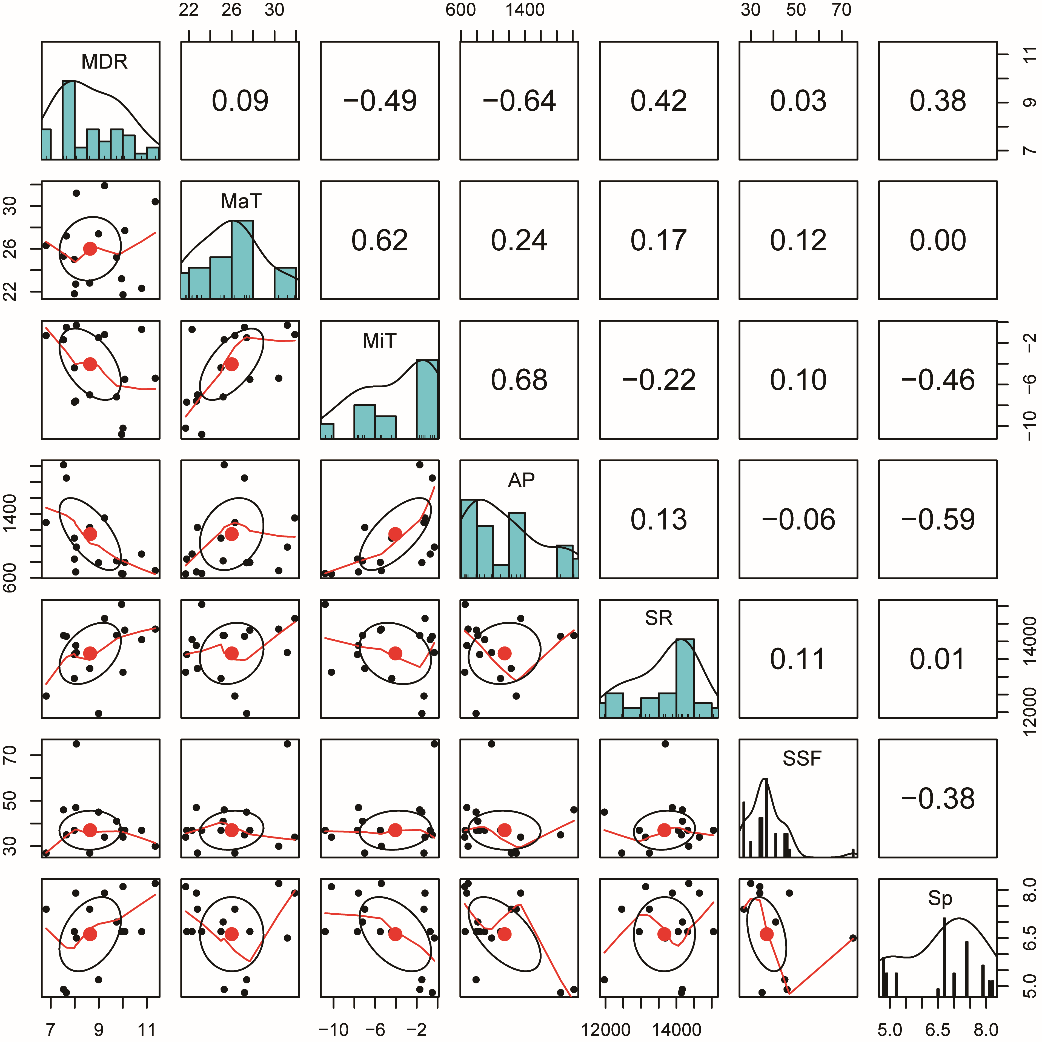


**Figure S14. Correlations among environment predictors after filtrating.** The lower left shows scatter plots and an ellipse around the mean with the axis length reflecting one standard deviation of the x and y variables. The diagonal shows histograms of the data and the lowess locally fit regression line as well. The higher right shows the Pearson correlation.


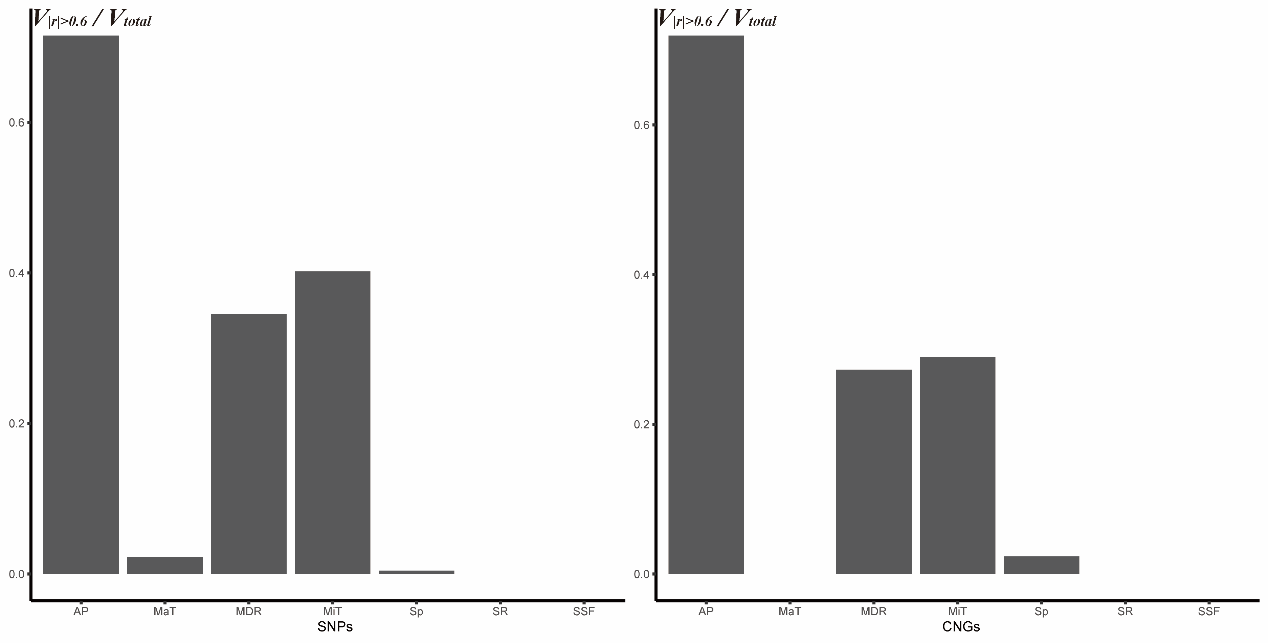


**Figure S15. Statistics of Variations (SNPs and CNGs) highly correlated with environmental predictors.** The vertical axis represents the ratio of the variations that are highly correlated (|r|>0.6) with the environments to the overall variations.


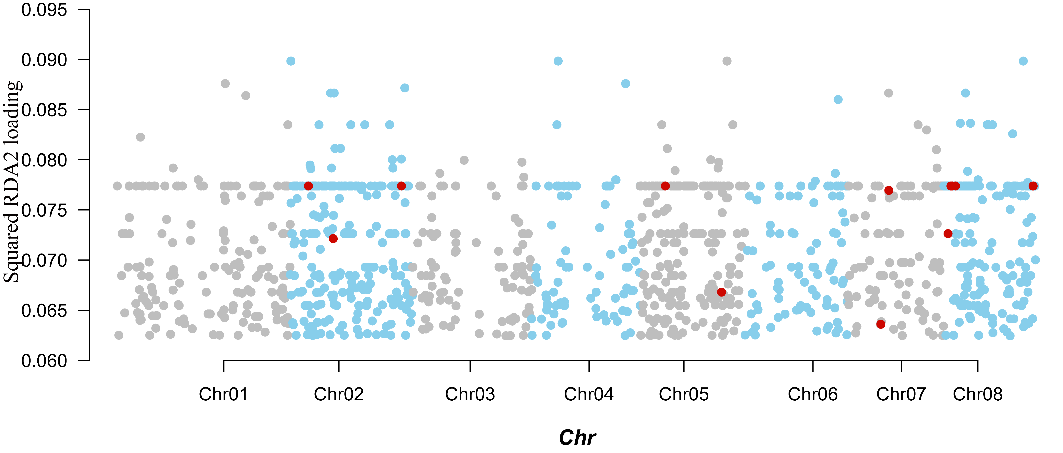


**Figure S16. Distributions of outlier SNPs (defined by RDA) on the second constrained axes (RDA2).** The red points represent variations inside the genomic islands.


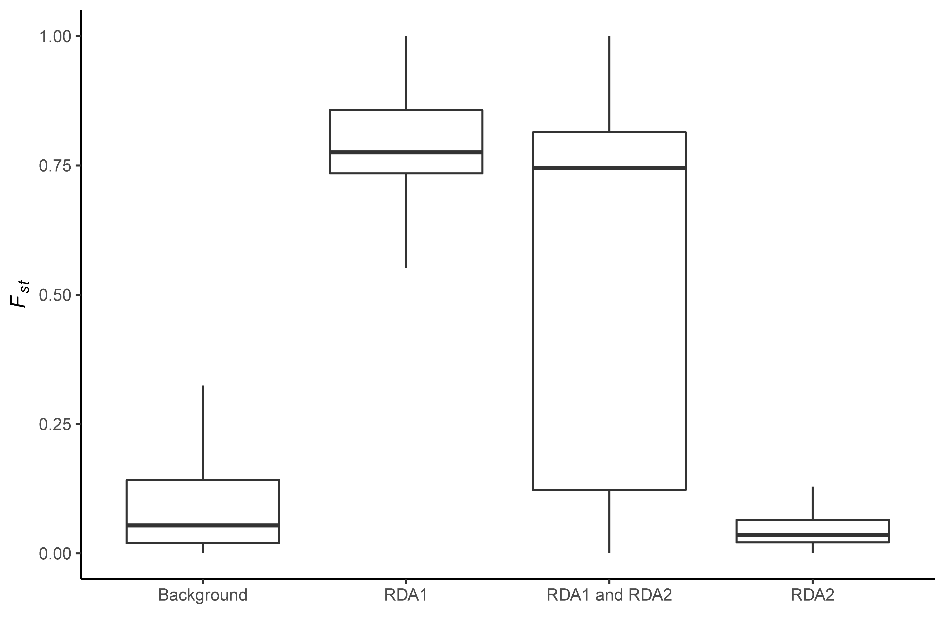


**Figure S17. Boxplot about changes in *F*_ST_ of outlier SNPs from RDA compared to background *F*_ST_.**

**
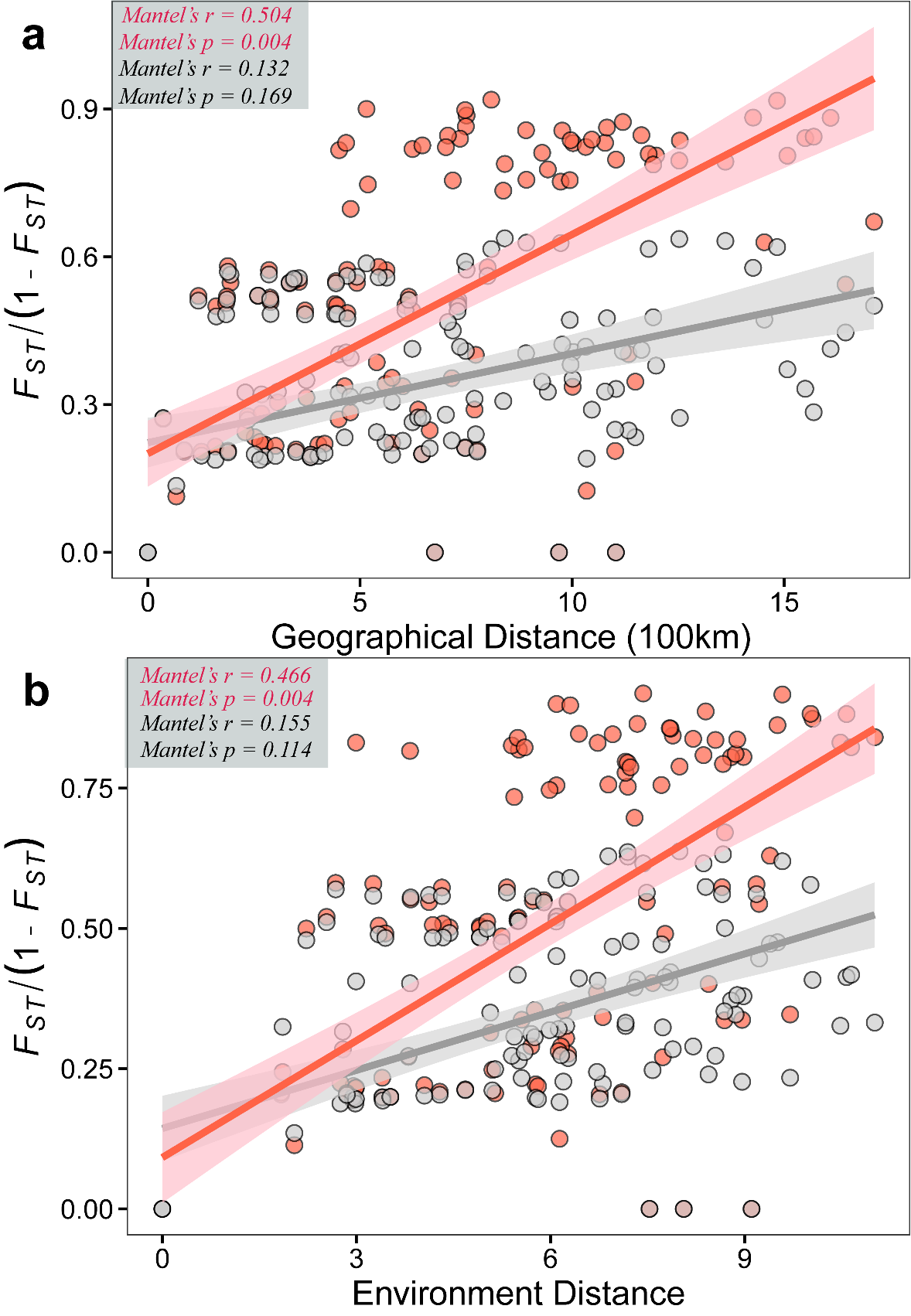
**

**Figure S18. Isolation analyses based on adptive SNPs and neutral SNPs.** (a) and (b) Isolation-by-distance and Isolation-by-environment. The shadow of linear regression denotes the 95% confidence interval. Red refers to adptive SNP datasets, gray refers to neutral SNP datasets.

**Table S1. Overview of sample information and sequencing statistics.**

| **Sample ID** | **Species** | **Description** | **Clean Reads (Gbp)** | **Map Ratio (%)** | **Genome Coverage (%)** | **Effective Depth** |
| --- | --- | --- | --- | --- | --- | --- |
| Och01 | *Ostrya chinensis* | Zehei Township, Kunming City, Yunnan Province, China | 13.6 | 0.94 | 0.93 | 66.73 |
| Och02 | *Ostrya chinensis* | Shiyang Forestry District, Wenzhou City, Zhejiang Province, China | 4.9 | 0.91 | 0.90 | 17.40 |
| Och03 | *Ostrya chinensis* | Shiyang Forestry District, Wenzhou City, Zhejiang Province, China | 4.8 | 0.93 | 0.90 | 14.48 |
| Och04 | *Ostrya chinensis* | Shiyang Forestry District, Wenzhou City, Zhejiang Province, China | 12.3 | 0.89 | 0.92 | 36.38 |
| Och05 | *Ostrya chinensis* | Shiyang Forestry District, Wenzhou City, Zhejiang Province, China | 10.5 | 0.86 | 0.92 | 29.99 |
| Och06 | *Ostrya chinensis* | Shiyang Forestry District, Wenzhou City, Zhejiang Province, China | 11.7 | 0.89 | 0.91 | 32.06 |
| Och07 | *Ostrya chinensis* | Fanjing Mountain, Tongren City, Guizhou Province, China | 4.7 | 0.90 | 0.90 | 14.15 |
| Och08 | *Ostrya chinensis* | Fanjing Mountain, Tongren City, Guizhou Province, China | 4 | 0.86 | 0.89 | 11.61 |
| Och09 | *Ostrya chinensis* | Fanjing Mountain, Tongren City, Guizhou Province, China | 5.3 | 0.90 | 0.90 | 16.24 |
| Och10 | *Ostrya chinensis* | Fanjing Mountain, Tongren City, Guizhou Province, China | 13.1 | 0.91 | 0.92 | 40.58 |
| Och11 | *Ostrya chinensis* | Fanjing Mountain, Tongren City, Guizhou Province, China | 11.2 | 0.92 | 0.92 | 35.88 |
| Och12 | *Ostrya chinensis* | Longquan Mountain, Lishui City, Zhejiang Province, China | 2.9 | 0.95 | 0.87 | 9.74 |
| Och13 | *Ostrya chinensis* | Longquan Mountain, Lishui City, Zhejiang Province, China | 2.9 | 0.95 | 0.87 | 10.90 |
| Och14 | *Ostrya chinensis* | Longquan Mountain, Lishui City, Zhejiang Province, China | 2.9 | 0.92 | 0.88 | 9.49 |
| Oja01 | *Ostrya japonica* | Shennongjia Forestry District, Hubei Province, China | 11.1 | 0.90 | 0.92 | 39.44 |
| Oja02 | *Ostrya japonica* | Shennongjia Forestry District, Hubei Province, China | 5.6 | 0.90 | 0.91 | 20.08 |
| Oja03 | *Ostrya japonica* | Shennongjia Forestry District, Hubei Province, China | 5.2 | 0.86 | 0.90 | 17.16 |
| Oja04 | *Ostrya japonica* | Ningshan County, Ankang City, Shaanxi Province, China | 4.7 | 0.92 | 0.90 | 16.91 |
| Oja05 | *Ostrya japonica* | Ningshan County, Ankang City, Shaanxi Province, China | 11.1 | 0.92 | 0.92 | 34.92 |
| Oja06 | *Ostrya japonica* | Ningshan County, Ankang City, Shaanxi Province, China | 4.8 | 0.91 | 0.90 | 16.79 |
| Oja07 | *Ostrya japonica* | Zhouqu County, Gansu Province, China | 3.7 | 0.81 | 0.89 | 9.98 |
| Oja08 | *Ostrya japonica* | Zhouqu County, Gansu Province, China | 14.8 | 0.75 | 0.92 | 26.85 |
| Oja09 | *Ostrya japonica* | Pingwu County, Mianyang City, Sichuan Province, China | 2.9 | 0.84 | 0.88 | 8.50 |
| Oja10 | *Ostrya japonica* | Pingwu County, Mianyang City, Sichuan Province, China | 4.7 | 0.90 | 0.91 | 16.40 |
| Oja11 | *Ostrya japonica* | Langao County, Ankang City, Shanxi Province, China | 5 | 0.83 | 0.90 | 16.05 |
| Oja12 | *Ostrya japonica* | Langao County, Ankang City, Shanxi Province, China | 4.4 | 0.90 | 0.90 | 15.91 |
| Oja13 | *Ostrya japonica* | Song County, Luoyang City, Henan Province, China | 4.3 | 0.92 | 0.90 | 12.24 |
| Oja14 | *Ostrya japonica* | Song County, Luoyang City, Henan Province, China | 4.3 | 0.92 | 0.90 | 12.32 |
| Oja15 | *Ostrya japonica* | Mount Li, Jincheng City, Shanxi Province, China | 12.9 | 0.93 | 0.93 | 40.24 |
| Oja16 | *Ostrya japonica* | Mount Li, Jincheng City, Shanxi Province, China | 4.4 | 0.93 | 0.90 | 13.42 |
| Oja17 | *Ostrya japonica* | Dakuaidi Primeval Forest Scenic, Sanmenxia City, Henan Province, China | 12 | 0.93 | 0.92 | 37.01 |
| Oja18 | *Ostrya japonica* | Dakuaidi Primeval Forest Scenic, Sanmenxia City, Henan Province, China | 4.7 | 0.92 | 0.91 | 14.33 |
| Oja19 | *Ostrya japonica* | Ta-pieh Mountains, Liuan City, Anhui Province, China | 5.5 | 0.88 | 0.91 | 18.90 |
| Oja20 | *Ostrya japonica* | Ta-pieh Mountains, Liuan City, Anhui Province, China | 11.4 | 0.82 | 0.92 | 30.56 |
| Oja21 | *Ostrya japonica* | Ta-pieh Mountains, Liuan City, Anhui Province, China | 4.7 | 0.83 | 0.90 | 15.30 |
| Oja23 | *Ostrya japonica* | Dangchuan Town, Tianshui City, Gansu Province, China | 7.5 | 0.91 | 0.91 | 27.22 |
| Oja24 | *Ostrya japonica* | Jiaohe Town, Luoyang City, Henan Province, China | 2.9 | 0.85 | 0.88 | 8.47 |
| Oja25 | *Ostrya japonica* | Jiaohe Town, Luoyang City, Henan Province, China | 2.9 | 0.87 | 0.88 | 8.79 |
| Oja26 | *Ostrya japonica* | Jiaohe Town, Luoyang City, Henan Province, China | 5 | 0.87 | 0.90 | 17.06 |
| Oja27 | *Ostrya japonica* | Pingwu County, Mianyang City, Sichuan Province, China | 2.9 | 0.93 | 0.88 | 10.70 |

**Table S2. *Tracy-Widom* (TW) statistics and P-values of the first ten eigenvalues in PCA*.***

| **Number** | **eigenvalue** | **TWstat** | **P-value** |
| --- | --- | --- | --- |
| 1 | 19.164105 | 2.849 | 0.0022786 |
| 2 | 1.786535 | 11.688 | 2.06E-13 |
| 3 | 1.211584 | 7.433 | 4.10E-08 |
| 4 | 0.945372 | 2.817 | 0.00241874 |
| 5 | 0.824254 | 0.189 | 0.136241 |
| 6 | 0.749789 | -1.589 | 0.601932 |
| 7 | 0.662111 | -4.708 | 0.999173 |
| 8 | 0.654889 | -4.292 | 0.996743 |
| 9 | 0.623528 | -5.092 | 0.999807 |
| 10 | 0.590727 | -6.09 | 0.999998 |

**Table S3. Relative likelihood of the different models shown in Figure S4.**

| **Model** | **best_likelhood** | ***AIC*** | ***ΔAIC*** | ***wiAIC*** |
| --- | --- | --- | --- | --- |
| model0 | -41,300,894.21 | 190,197,654.66 | 1,344,849.49 | 0 |
| model1 | -41,061,714.93 | 189,096,197.39 | 243,392.22 | 0 |
| model2 | -41,017,432.06 | 188,892,275.21 | 39,470.03 | 0 |
| model3 | -41,028,585.55 | 188,943,638.94 | 90,833.77 | 0 |
| model4 | -41,019,019.42 | 188,899,593.30 | 46,788.12 | 0 |
| model5 | -41,010,888.98 | 188,862,151.24 | 9,346.07 | 0 |
| model6 | -41,011,155.98 | 188,863,388.81 | 10,583.63 | 0 |
| model7 | -41,013,085.57 | 188,872,274.90 | 19,469.73 | 0 |
| model8 | -41,013,465.20 | 188,874,023.14 | 21,217.97 | 0 |
| model9 | -41,019,205.16 | 188,900,456.66 | 47,651.49 | 0 |
| model10 | -41,008,928.89 | 188,853,140.68 | 335.51 | 1.39959E-73 |
| **model11** | **-41,008,856.04** | **188,852,805.17** | **0.00** | **1** |
| model12 | -41,012,769.15 | 188,870,825.73 | 18,020.56 | 0 |
| model13 | -41,017,488.66 | 188,892,559.90 | 39,754.73 | 0 |

**Table S4. Inferred parameters estimates with 95% confidence intervals for the best fitting demographic scenario modelled in fastsimcoal2, with parameter tags corresponding to the model shown in Figure S5.**

| **Parameters** | **Point estimate** | **95% confidence intervals** | |
| --- | --- | --- | --- |
|  |  | **Lower bound** | **Upper bound** |
| NPOP0 | 58,337 | 56,270 | 60,403 |
| NPOP02 | 24,938 | 18,669 | 31,208 |
| NPOP03 | 543,344 | 408,798 | 677,890 |
| NPOP1 | 102,708 | 100,605 | 104,810 |
| NPOP12 | 49,695 | 36,680 | 62,711 |
| NPOP13 | 498,220 | 365,466 | 630,974 |
| NPOP | 423,871 | 300,474 | 547,268 |
| TH1. | 430,314 | 373,743 | 486,884 |
| TH2. | 634,460 | 534,356 | 734,563 |
| TH3. | 1,495,758 | 1,066,137 | 1,925,378 |
| TH4. | 2,584,553 | 2,173,844 | 2,995,261 |
| TH5. | 3,742,475 | 3,056,762 | 4,428,187 |
| MR0M01 | 8.35E-07 | 7.51E-07 | 9.19E-07 |
| MR0M10 | 7.27E-07 | 6.80E-07 | 7.75E-07 |
| MR1M01 | 1.66E-05 | 9.49E-06 | 2.37E-05 |
| MR1M10 | 1.40E-06 | 8.45E-07 | 1.95E-06 |
| MR2M01 | 6.54E-06 | 2.96E-06 | 1.01E-05 |
| MR2M10 | 2.20E-06 | 8.17E-07 | 3.59E-06 |
| MR3M01 | 3.29E-06 | 4.68E-08 | 6.54E-06 |
| MR3M10 | 3.01E-05 | 0.00E+00 | 6.52E-05 |
| MR4M01 | 7.72E-07 | 3.03E-07 | 1.24E-06 |
| MR4M10 | 2.61E-06 | 0.00E+00 | 5.35E-06 |

**Table S5. Geographical distribution of *O. japonica* and *O. chinensis*.**

| **species** | **longitude** | **latitude** |
| --- | --- | --- |
| *O. japonica* | 110.4356 | 31.67904 |
| *O. japonica* | 108.504 | 33.54712 |
| *O. japonica* | 104.3697 | 33.58554 |
| *O. japonica* | 104.4043 | 32.44692 |
| *O. japonica* | 108.8138 | 32.13428 |
| *O. japonica* | 112.2625 | 33.78198 |
| *O. japonica* | 112.0048 | 35.4326 |
| *O. japonica* | 110.9947 | 33.97339 |
| *O. japonica* | 116.3313 | 31.4072 |
| *O. japonica* | 106.2037 | 34.33159 |
| *O. japonica* | 111.338 | 33.81906 |
| *O. japonica* | 104.4043 | 32.44691 |
| *O. japonica* | 114.0764 | 32.11319 |
| *O. japonica* | 117.5203 | 40.43602 |
| *O. japonica* | 106.665 | 35.543 |
| *O. japonica* | 110.6647 | 31.75861 |
| *O. japonica* | 113.9787 | 37.40295 |
| *O. japonica* | 107.8917 | 34.09167 |
| *O. japonica* | 107.9554 | 33.69386 |
| *O. japonica* | 108.4511 | 33.43552 |
| *O. japonica* | 109.0889 | 32.25065 |
| *O. japonica* | 112.086 | 34.1344 |
| *O. japonica* | 112.429 | 33.48986 |
| *O. japonica* | 111.616 | 33.7856 |
| *O. japonica* | 111.8468 | 34.02312 |
| *O. japonica* | 110.8318 | 33.75653 |
| *O. japonica* | 107.319 | 34.05847 |
| *O. japonica* | 108.314 | 33.31079 |
| *O. japonica* | 108.605 | 34.1087 |
| *O. japonica* | 108.902 | 32.30719 |
| *O. chinensis* | 102.6896 | 26.13741 |
| *O. chinensis* | 119.8414 | 27.8703 |
| *O. chinensis* | 108.6485 | 27.92109 |
| *O. chinensis* | 119.1589 | 27.87167 |
| *O. chinensis* | 119.9239 | 29.6212 |
| *O. chinensis* | 110.857 | 26.43365 |
| *O. chinensis* | 111.139 | 29.42991 |
| *O. chinensis* | 112.949 | 25.3998 |
| *O. chinensis* | 109.7402 | 26.25781 |
| *O. chinensis* | 108.9359 | 30.29124 |
| *O. chinensis* | 110.5522 | 30.08367 |
| *O. chinensis* | 108.5258 | 27.99295 |
| *O. chinensis* | 108.1241 | 27.03328 |
| *O. chinensis* | 108.4098 | 27.9932 |
| *O. chinensis* | 109.784 | 26.15799 |
| *O. chinensis* | 111.38 | 29.58429 |
| *O. chinensis* | 110.1579 | 29.39518 |
| *O. chinensis* | 109.4792 | 30.29514 |
| *O. chinensis* | 119.8525 | 27.81824 |
| *O. chinensis* | 108.6979 | 27.90777 |
| *O. chinensis* | 119.142 | 28.07426 |

**Table S6. Inferred parameters estimates with 95% confidence intervals for population genomic parameters along whole genome.**

|  | ***π*** | ***ρ*** | ***F*_ST_** | ***D*_xy_** |
| --- | --- | --- | --- | --- |
| *O. japonica* | 0.001260801  ±8.314e-06 | 69.62666  ±0.53769 | 0.3471887  ±0.0011554 | 0.004585667  ±2.335e-05 |
| *O. chinensis* | 0.001032443  ±7.517e-06 | 14.99125  ±0.18308 |  |  |
| P-value | < 2.2e-16 | < 2.2e-16 | - | - |

**Table S7. Distribution of islands on each chromosome.**

| **Chromosome** | **Island windows distributed over one chromosome** | **windows distributed over one chromosome** | **all island windows** | **all windows** | **P-value** |
| --- | --- | --- | --- | --- | --- |
| Chr01 | 72 | 11870 | 607 | 60755 | 6.90E-05 |
| Chr02 | 35 | 7600 | 607 | 60755 | 7.04E-06 |
| Chr03 | 114 | 7846 | 607 | 60755 | 0.0003107 |
| Chr04 | 167 | 7298 | 607 | 60755 | 8.28E-22 |
| Chr05 | 38 | 6820 | 607 | 60755 | 0.0005292 |
| Chr06 | 41 | 6821 | 607 | 60755 | 0.0018892 |
| Chr07 | 25 | 6497 | 607 | 60755 | 1.79E-06 |
| Chr08 | 115 | 6003 | 607 | 60755 | 1.65E-10 |

**Table S8. GO enrichment results of positive selection genes identified by HKA test.**

| **GO_ID** | **Class** | **Term** | **Adjust *P* value** |
| --- | --- | --- | --- |
| ***O. japonica*** |  |  |  |
| GO:0016887 | Molecular Function | ATP hydrolysis activity | 0.044251142 |
| GO:0017111 | Molecular Function | nucleoside-triphosphatase activity | 0.044251142 |
| GO:0050321 | Molecular Function | tau-protein kinase activity | 0.044251142 |
| GO:0004435 | Molecular Function | phosphatidylinositol phospholipase C activity | 0.044251142 |
| GO:0046777 | Biological Process | protein autophosphorylation | 0.002120408 |
| GO:0010073 | Biological Process | meristem maintenance | 0.002332525 |
| GO:0006302 | Biological Process | double-strand break repair | 0.002332525 |
| GO:0009908 | Biological Process | flower development | 0.002332525 |
| GO:0016569 | Biological Process | obsolete covalent chromatin modification | 0.002332525 |
| GO:0009873 | Biological Process | ethylene-activated signaling pathway | 0.003401685 |
| GO:0006338 | Biological Process | chromatin remodeling | 0.00428401 |
| GO:0005982 | Biological Process | starch metabolic process | 0.008526335 |
| GO:0006310 | Biological Process | DNA recombination | 0.018990115 |
| GO:0006812 | Biological Process | cation transport | 0.042218509 |
| ***O. chinensis*** |  |  |  |
| GO:0005451 | Molecular Function | monovalent cation:proton antiporter activity | 0.001259601 |
| GO:0050321 | Molecular Function | tau-protein kinase activity | 0.026359587 |
| GO:0004386 | Molecular Function | helicase activity | 0.027515969 |
| GO:0016887 | Molecular Function | ATP hydrolysis activity | 0.031402381 |
| GO:0050660 | Molecular Function | flavin adenine dinucleotide binding | 0.044940251 |
| GO:0002161 | Molecular Function | aminoacyl-tRNA editing activity | 0.044940251 |
| GO:0070062 | Cellular Component | extracellular exosome | 0.008679926 |
| GO:0005871 | Cellular Component | kinesin complex | 0.024415675 |
| GO:0016569 | Biological Process | obsolete covalent chromatin modification | 6.80E-05 |
| GO:0009908 | Biological Process | flower development | 0.000409269 |
| GO:0006302 | Biological Process | double-strand break repair | 0.003733844 |
| GO:0006310 | Biological Process | DNA recombination | 0.003800793 |

**Table S9. Positive selection genes that located in the genomic islands.**

| **Gene ID** | **P-value of HKA test for**  ***O. japonica*** | **P-value of HKA test for**  ***O. chinensis*** | **Unique and stable entry identifier in UniProtKB** | **Gene name** |
| --- | --- | --- | --- | --- |
| OreG0000089.2 | 1.34E-16 | 5.63E-21 | - | - |
| OreG0000137.3 | 2.08E-08 | 5.06E-06 | - | - |
| OreG0000138.2 | 5.42E-08 | 1.89E-07 | - | - |
| OreG0000139.2 | 0.000312688 | 0.002095865 | - | - |
| OreG0000145.2 | - | 0.004930204 | - | - |
| OreG0000683.1 | 0.009291848 | - | - | - |
| OreG0000785.3 | 0.000163309 | 0.004880421 | Q9ZRF1 | CAD |
| OreG0002377.1 | 0.003333653 | 0.004930204 | - | - |
| OreG0002379.1 | 0.001209962 | 0.00843184 | - | - |
| OreG0003056.2 | 4.38E-43 | 2.81E-28 | - | - |
| OreG0003057.1 | - | 0.00192045 | - | - |
| OreG0003058.4 | 8.42E-06 | 1.90E-05 | Q06942 | - |
| OreG0004463.1 | 5.89E-10 | 1.91E-11 | - | - |
| OreG0004465.1 | 1.19E-06 | 6.75E-05 | Q9LQI9 | UBP1B |
| OreG0005007.1 | 0.000443009 | - | - | - |
| OreG0006134.1 | 0.000119309 | 0.000236798 | - | - |
| OreG0006136.3 | 0.002968735 | 0.000298548 | - | - |
| OreG0006257.1 | 0.009018963 | - | P50287 | At5g08100 |
| OreG0006259.1 | 3.55E-05 | 8.67E-07 | - | - |
| OreG0008316.1 | 2.53E-05 | 2.39E-06 | - | - |
| OreG0008318.1 | - | 0.000578865 | - | - |
| OreG0008520.3 | 0.000443009 | 0.000754624 | O04714 | GCR1 |
| OreG0009255.1 | 2.70E-08 | 2.61E-08 | - | - |
| OreG0009256.1 | 0.000474658 | 0.000236798 | - | - |
| OreG0010184.2 | 0.001508955 | 4.75E-05 | - | - |
| OreG0010185.2 | 0.000626322 | 0.000379208 | - | - |
| OreG0010232.1 | 0.003333653 | 0.004930204 | - | - |
| OreG0010236.4 | 0.002968735 | 0.001414927 | Q8GZQ3 | PNP1 |
| OreG0010677.3 | 1.80E-07 | 8.85E-10 | - | - |
| OreG0011042.1 | 0.009291848 | - | - | - |
| OreG0011199.1 | 1.43E-09 | 2.06E-07 | - | - |
| OreG0011200.1 | 0.001209962 | - | - | - |
| OreG0011204.1 | 0.003333653 | - | - | - |
| OreG0011318.1 | 0.003333653 | - | - | - |
| OreG0011474.1 | 3.16E-06 | - | - | - |
| OreG0011476.2 | 0.002144668 | - | - | - |
| OreG0011811.1 | 0.000119309 | 0.00089386 | E0CTY1 | GATA |
| OreG0012742.1 | 3.49E-09 | 5.06E-06 | - | - |
| OreG0013067.3 | 0.000819255 | 0.000298548 | - | - |
| OreG0013068.5 | 0.009018963 | 0.000578865 | Q75HX5 | TULP8 |
| OreG0013232.1 | 6.61E-11 | 1.88E-09 | - | - |
| OreG0013233.5 | 0.009291848 | - | Q9SZ96 | URGT5 |
| OreG0013238.2 | 2.42E-08 | 0.000203877 | - | - |
| OreG0014052.1 | 0.003333653 | - | - | - |
| OreG0014239.2 | 4.58E-14 | 1.07E-16 | - | - |
| OreG0014592.1 | 0.001191446 | - | - | - |
| OreG0014978.1 | - | 4.33E-05 | - | - |
| OreG0014979.1 | - | 0.00192045 | - | - |
| OreG0015035.1 | 0.000257506 | 3.97E-05 | - | - |
| OreG0015807.1 | 2.91E-05 | 3.09E-06 | - | - |
| OreG0015811.1 | 0.005604418 | 0.00843184 | - | - |
| OreG0015813.1 | 6.89E-06 | 2.06E-07 | - | - |
| OreG0015817.1 | 4.26E-08 | 3.88E-10 | - | - |
| OreG0016342.1 | 0.002968735 | 0.004880421 | - | - |
| OreG0016343.4 | 0.009291848 | - | - | - |
| OreG0016458.1 | 0.007325405 | - | - | - |
| OreG0016459.2 | 0.000163309 | 0.004880421 | - | - |
| OreG0016460.1 | 4.55E-05 | 9.69E-05 | - | - |
| OreG0016462.1 | 1.14E-05 | 6.65E-06 | - | - |
| OreG0017482.1 | 4.41E-13 | 4.57E-12 | - | - |
| OreG0017846.1 | 0.003593039 | 0.000118759 | Q9FN03 | UVR8 |
| OreG0017847.2 | - | 0.000578865 | - | - |
| OreG0017958.1 | 0.005604418 | 0.00192045 | - | - |
| OreG0017959.1 | 0.007325405 | 0.000754624 | - | - |
| OreG0018317.1 | 0.001209962 | 0.00192045 | - | - |
| OreG0018318.1 | 0.000312688 | 0.000578865 | - | - |
| OreG0018678.1 | 6.61E-11 | 1.88E-09 | - | - |
| OreG0019036.1 | 6.05E-05 | - | O49354 | COQ3 |
| OreG0019042.1 | 0.009291848 | - | - | - |
| OreG0019267.3 | 0.000446707 | 1.86E-05 | Q9ZVF7 | ESMD1 |
| OreG0019269.1 | 0.009291848 | - | - | - |
| OreG0019554.1 | 1.03E-07 | 7.75E-08 | - | - |
| OreG0019562.4 | 0.003333653 | - | - | - |
| OreG0019633.2 | 2.75E-06 | 2.64E-05 | - | - |
| OreG0020010.2 | 3.16E-06 | 3.97E-05 | Q9MAU6 | PDIL2-2 |
| OreG0020554.1 | 1.19E-06 | 3.09E-06 | - | - |
| OreG0020691.1 | 0.003333653 | 0.004930204 | - | - |
| OreG0021360.2 | 2.75E-06 | 7.75E-05 | - | - |
| OreG0021463.3 | 6.92E-14 | 3.35E-12 | - | - |
| OreG0021487.1 | 3.16E-06 | - | - | - |
| OreG0021608.3 | 4.49E-10 | 5.40E-14 | - | - |
| OreG0022200.1 | 0.000203569 | 0.000487137 | - | - |
| OreG0022201.1 | 1.64E-09 | 1.15E-07 | - | - |
| OreG0022266.1 | 0.000163309 | 0.004880421 | - | - |
| OreG0022267.1 | 0.001209962 | 0.00843184 | - | - |
| OreG0022268.1 | 4.47E-07 | 1.25E-06 | - | - |
| OreG0022490.1 | 6.89E-06 | 1.86E-05 | - | - |
| OreG0022727.1 | 0.001209962 | 0.00192045 | - | - |
| OreG0022728.1 | 2.34E-05 | 1.86E-05 | - | - |
| OreG0022786.4 | 0.000819255 | 0.004880421 | - | - |
| OreG0022787.1 | 1.75E-13 | 4.65E-13 | - | - |
| OreG0024274.3 | 1.07E-11 | 3.25E-08 | - | - |
| OreG0024285.1 | 0.001508955 | 0.000236798 | - | - |
| OreG0024545.1 | 4.21E-41 | 2.50E-31 | Q9SIB9 | ACO3 |
| OreG0024661.3 | 1.93E-10 | 5.39E-09 | - | - |
| OreG0024872.5 | 6.99E-26 | 1.83E-25 | - | - |
| OreG0025137.1 | 0.00175202 | 9.69E-05 | - | - |
| OreG0026366.1 | 6.89E-06 | 0.000170524 | Q9ZR72 | ABCB1 |
| OreG0026684.2 | 0.003333653 | 0.004930204 | - | - |
| OreG0026709.1 | 2.65E-07 | 3.50E-05 | - | - |
| OreG0026743.3 | 4.47E-07 | 1.25E-06 | - | - |
| OreG0027028.1 | 2.25E-05 | 4.75E-05 | - | - |
| OreG0027358.4 | - | 0.004930204 | - | - |
| OreG0027359.1 | 6.51E-07 | 3.18E-08 | - | - |
| OreG0027361.1 | 7.37E-16 | 9.43E-11 | - | - |

**Table S10. GO enrichment results of positive selection genes that located in genomic islands.**

| **GO_ID** | **Class** | **Term** | **Adjust *P* value** |
| --- | --- | --- | --- |
| GO:0009411 | Biological Process | response to UV | 0.006119919 |
| GO:0006281 | Biological Process | DNA repair | 0.006119919 |
| GO:0007166 | Biological Process | cell surface receptor signaling pathway | 0.018408762 |

**Table S11. GO enrichment results of CNDGs between *O. japonica* and *O. chinensis***

| **GO_ID** | **Class** | **Term** | **Adjust *P* value** |
| --- | --- | --- | --- |
| GO:0102580 | Molecular Function | cyanidin 3-O-glucoside 2-O''-xylosyltransferase activity | 0.001533857 |
| GO:0030246 | Molecular Function | carbohydrate binding | 0.001894411 |
| GO:0008171 | Molecular Function | O-methyltransferase activity | 0.001894411 |
| GO:0004568 | Molecular Function | chitinase activity | 0.001894411 |
| GO:0005234 | Molecular Function | extracellularly glutamate-gated ion channel activity | 0.004649057 |
| GO:0004970 | Molecular Function | ionotropic glutamate receptor activity | 0.00492144 |
| GO:0008199 | Molecular Function | ferric iron binding | 0.005675772 |
| GO:0008757 | Molecular Function | S-adenosylmethionine-dependent methyltransferase activity | 0.010207433 |
| GO:0043531 | Molecular Function | ADP binding | 0.010229948 |
| GO:0050662 | Molecular Function | obsolete coenzyme binding | 0.017008608 |
| GO:0047672 | Molecular Function | anthranilate N-benzoyltransferase activity | 0.024178583 |
| GO:0006032 | Biological Process | chitin catabolic process | 0.000145593 |
| GO:0019438 | Biological Process | aromatic compound biosynthetic process | 0.000666671 |
| GO:0035235 | Biological Process | ionotropic glutamate receptor signaling pathway | 0.011446158 |
| GO:0009646 | Biological Process | response to absence of light | 0.020754589 |
| GO:0009699 | Biological Process | phenylpropanoid biosynthetic process | 0.031130299 |
| GO:0006880 | Biological Process | intracellular sequestering of iron ion | 0.031130299 |
| GO:0000288 | Biological Process | nuclear-transcribed mRNA catabolic process, deadenylation-dependent decay | 0.038770611 |
| GO:0006826 | Biological Process | iron ion transport | 0.039013643 |
| GO:0009809 | Biological Process | lignin biosynthetic process | 0.044827072 |

**Table S12. The proportion of the variance explained and *P* value for each constrained axes in RDA analyses.**

| **constrained axes** | **Adjusted Proportion Explained** | | **Adjusted Cumulative Proportion** | | **P-value** | |
| --- | --- | --- | --- | --- | --- | --- |
|  | **SNPs** | **CNGs** | **SNPs** | **CNGs** | **SNPs** | **CNGs** |
| RDA1 | 0.033734037 | 0.043678237 | 0.033734037 | 0.04367824 | 0.001 | 0.001 |
| RDA2 | 0.014623673 | 0.013107275 | 0.04835771 | 0.05678551 | 0.002 | 0.488 |
| RDA3 | 0.01260047 | 0.012218244 | 0.06095818 | 0.06900376 | 0.063 | 0.48 |
| RDA4 | 0.011981978 | 0.009252083 | 0.072940157 | 0.07825584 | 0.319 | 0.893 |
| RDA5 | 0.011908597 | 0.008204345 | 0.084848754 | 0.08646018 | 0.291 | 0.966 |
| RDA6 | 0.010447278 | 0.007553078 | 0.095289743 | 0.09401326 | 0.541 | 0.951 |
| RDA7 | 0.009537361 | 0.0072199 | 0.1048292 | 0.10123316 | 0.362 | 0.807 |

**Table S13. 95% confidence intervals for environment predictors.**

|  | **Mean Diurnal Range (MDR)(°C)** | **Max Temperature (MaT)(°C)** | **Min Temperature (MiT)(°C)** | **Annual Precipitation (AP) (mm)** | **Solar Radiation (SR)(kJ m-2 day-1)** | **Subsoil Sand Fraction (SSF)(% weight)** | **Subsoil pH (Sp)(-log(H+))** |
| --- | --- | --- | --- | --- | --- | --- | --- |
| *O. japonica* | 9.230128  ±0.4032475 | 25.93846  ±1.450975 | -5.665385  ±1.314284 | 893.3462  ±95.27788 | 13726.56  ±360.0437 | 38.34615  ±3.684112 | 7.015385  ±0.3579447 |
| *O. chinensis* | 7.548214  ±0.5830309 | 26.12143  ±0.7613439 | -1.057143  ±0.2871008 | 1617.643  ±214.2999 | 13540.07  ±483.6724 | 34.64286  ±4.191847 | 5.885714  ±0.7327417 |
| P-value | 2.85E-05 | 0.8177 | 1.30E-07 | 2.61E-06 | 0.5168 | 0.1701 | 0.007668 |
| sd(Oja)/sd(Omu) | 0.9886912 | 2.72433 | 6.543882 | 0.6355515 | 1.064105 | 1.256343 | 0.6983058 |

**Table S14. GO enrichment results of genes with outlier SNPs identified by RDA analyses.**

| **GO_ID** | **Class** | **Term** | **Adjust *P* value** |
| --- | --- | --- | --- |
| GO:0008094 | Molecular Function | ATP-dependent activity, acting on DNA | 0.035268611 |
| GO:0044464 | Cellular Component | obsolete cell part | 0.016784791 |
| GO:0016604 | Cellular Component | nuclear body | 0.022393363 |
| GO:0005777 | Cellular Component | peroxisome | 0.03238255 |
| GO:0000145 | Cellular Component | exocyst | 0.039478467 |
| GO:0048471 | Cellular Component | perinuclear region of cytoplasm | 0.039478467 |
| GO:0005856 | Cellular Component | cytoskeleton | 0.039478467 |
| GO:0000775 | Cellular Component | chromosome, centromeric region | 0.039478467 |
| GO:0005694 | Cellular Component | chromosome | 0.039478467 |
| GO:0005666 | Cellular Component | RNA polymerase III complex | 0.039478467 |
| GO:0048856 | Biological Process | anatomical structure development | 1.25E-03 |
| GO:0006369 | Biological Process | termination of RNA polymerase II transcription | 1.98E-02 |
| GO:0048731 | Biological Process | system development | 1.98E-02 |
| GO:0006281 | Biological Process | DNA repair | 2.11E-02 |

**Table S15 GO enrichment results of outlier CNGs identified by RDA analyses.**

| **GO_ID** | **Class** | **Term** | **Adjust *P* value** |
| --- | --- | --- | --- |
| GO:0080074 | Molecular Function | spermidine:caffeoyl CoA N-acyltransferase activity | 0.000133237 |
| GO:0080075 | Molecular Function | spermidine:feruloyl CoA N-acyltransferase activity | 0.000133237 |
| GO:0005234 | Molecular Function | extracellularly glutamate-gated ion channel activity | 0.000133237 |
| GO:0080072 | Molecular Function | spermidine:sinapoyl CoA N-acyltransferase activity | 0.000133237 |
| GO:0080073 | Molecular Function | spermidine:coumaroyl CoA N-acyltransferase activity | 0.000133237 |
| GO:0043531 | Molecular Function | ADP binding | 0.000215308 |
| GO:0047672 | Molecular Function | anthranilate N-benzoyltransferase activity | 0.000258801 |
| GO:0004970 | Molecular Function | ionotropic glutamate receptor activity | 0.000265192 |
| GO:0102580 | Molecular Function | cyanidin 3-O-glucoside 2-O''-xylosyltransferase activity | 0.000385228 |
| GO:0050662 | Molecular Function | obsolete coenzyme binding | 0.000579338 |
| GO:0016410 | Molecular Function | N-acyltransferase activity | 0.003358586 |
| GO:0008199 | Molecular Function | ferric iron binding | 0.006450514 |
| GO:0008171 | Molecular Function | O-methyltransferase activity | 0.009030283 |
| GO:0045735 | Molecular Function | nutrient reservoir activity | 0.009183142 |
| GO:0004568 | Molecular Function | chitinase activity | 0.009183142 |
| GO:0016844 | Molecular Function | strictosidine synthase activity | 0.009522011 |
| GO:0030145 | Molecular Function | manganese ion binding | 0.009522011 |
| GO:0000175 | Molecular Function | 3'-5'-exoribonuclease activity | 0.026829306 |
| GO:0005545 | Molecular Function | 1-phosphatidylinositol binding | 0.02928255 |
| GO:0030246 | Molecular Function | carbohydrate binding | 0.031302664 |
| GO:0004715 | Molecular Function | non-membrane spanning protein tyrosine kinase activity | 0.038743056 |
| GO:0010334 | Molecular Function | sesquiterpene synthase activity | 0.041156838 |
| GO:0030276 | Molecular Function | clathrin binding | 0.043580406 |
| GO:0080088 | Biological Process | spermidine hydroxycinnamate conjugate biosynthetic process | 0.000240691 |
| GO:0035235 | Biological Process | ionotropic glutamate receptor signaling pathway | 0.001086884 |
| GO:0006032 | Biological Process | chitin catabolic process | 0.001419956 |
| GO:0009809 | Biological Process | lignin biosynthetic process | 0.001419956 |
| GO:0010584 | Biological Process | pollen exine formation | 0.002565732 |
| GO:0009699 | Biological Process | phenylpropanoid biosynthetic process | 0.005092284 |
| GO:0019438 | Biological Process | aromatic compound biosynthetic process | 0.005092284 |
| GO:0000288 | Biological Process | nuclear-transcribed mRNA catabolic process, deadenylation-dependent decay | 0.00806276 |
| GO:0000462 | Biological Process | maturation of SSU-rRNA from tricistronic rRNA transcript (SSU-rRNA, 5.8S rRNA, LSU-rRNA) | 0.010397555 |
| GO:0009646 | Biological Process | response to absence of light | 0.01552496 |
| GO:0033609 | Biological Process | oxalate metabolic process | 0.01552496 |
| GO:0006880 | Biological Process | intracellular sequestering of iron ion | 0.028485497 |
| GO:0006826 | Biological Process | iron ion transport | 0.043661205 |

**Table S16. The blast results of environmental adaptation genes.** (+ represents leaf-related genes, - represents root-related genes, * represents light-related genes)

| **Gene ID** | **Unique and stable entry identifier in UniProtKB** | **Gene name** | **% identity** | **alignment length** | **E-value** | **score** |
| --- | --- | --- | --- | --- | --- | --- |
| based on SNPs | |  |  |  |  |  |
| OreG0009806.1 | A9PCL4 | Peroxiredoxin-2 | 87.037 | 162 | 1.79E-100 | 288 |
| OreG0016281.1 | A4ZGQ8 | HMT1 | 80.183 | 328 | 0 | 559 |
| OreG0007389.3 | O82656 | PTP1 | 76.016 | 246 | 4.56E-144 | 411 |
| OreG0000026.1 | Q9SZ53 | At4g31860 | 80.791 | 354 | 0 | 612 |
| OreG0002732.1 | Q10MC0 | NIN1 | 80 | 505 | 0 | 863 |
| OreG0025474.1 | Q9ZNT1 | CBR1 | 80.952 | 252 | 1.56E-153 | 432 |
| OreG0002161.4 | Q07511 | FDH1 | 84.691 | 307 | 0 | 533 |
| OreG0019841.1 | P83373 | MMDHI | 85.549 | 346 | 0 | 595 |
| OreG0008245.3 | F4HXV6 | NUP155 | 75.268 | 1492 | 0 | 2221 |
| OreG0027072.1 | O64791 | SYP124 | 78.146 | 302 | 2.36E-174 | 487 |
| OreG0007194.3 | Q42541 | UBC13 | 90.826 | 109 | 3.72E-71 | 212 |
| OreG0017156.1 | Q9FM04 | At5g62930 | 76.446 | 242 | 2.83E-145 | 408 |
| OreG0004396.1 | O04130 | PGDH2 | 82.169 | 544 | 0 | 907 |
| OreG0020312.1 | Q42667 | PAL6 | 85.206 | 703 | 0 | 1250 |
| OreG0017796.6 | Q9C952 | CPSF73-I | 80.747 | 696 | 0 | 1199 |
| OreG0005949.1 | Q38931 | FKBP62 | 81.307 | 551 | 0 | 892 |
| OreG0002840.2 | Q9SCJ4 | KIN8B | 75.352 | 710 | 0 | 1068 |
| OreG0004683.1 | Q38884 | TIF3I1 | 75.908 | 303 | 2.53E-161 | 458 |
| OreG0019643.1 | Q84P54 | GABA-TP1 | 76.074 | 326 | 4.16E-176 | 504 |
| **OreG0006909.3+-*** | Q43125 | CRY1 | 76.648 | 531 | 0 | 749 |
| OreG0015872.1 | Q8W207 | CSN2 | 85.421 | 439 | 0 | 787 |
| OreG0020287.1 | Q9SP55 | VATG | 78.182 | 110 | 7.69E-57 | 174 |
| OreG0017295.1 | P37221 | NAD-dependent | 87.22 | 626 | 0 | 1113 |
| OreG0004111.1 | Q9ZUM2 | TOM3 | 84.698 | 281 | 0 | 505 |
| OreG0025435.1 | P27164 | CAM53 | 83.938 | 193 | 1.74E-110 | 316 |
| OreG0008302.1 | Q9LR75 | CPX1 | 86.61 | 351 | 0 | 630 |
| OreG0009462.2 | Q8LMR2 | DCL1 | 77.758 | 1677 | 0 | 2669 |
| **OreG0000976.1-** | Q9FPT1 | UBP12 | 83.062 | 1104 | 0 | 1897 |
| OreG0010091.3 | Q05728 | PUR5 | 82.677 | 254 | 1.30E-152 | 433 |
| OreG0001086.1 | Q96473 | V-type | 100 | 138 | 4.16E-91 | 264 |
| OreG0004782.1 | P53780 | At3g57050 | 76.407 | 462 | 0 | 740 |
| OreG0011893.1 | O22775 | XXT2 | 80.694 | 461 | 0 | 758 |
| OreG0026064.2 | A5A7I7 | CPK4 | 84.656 | 567 | 0 | 988 |
| **OreG0010074.1-** | Q9SRW9 | CSLD5 | 81.902 | 1199 | 0 | 1979 |
| OreG0012899.2 | Q680U9 | TFB2 | 80.563 | 355 | 0 | 602 |
| OreG0017996.1 | F4J3N2 | FTSHI5 | 75.415 | 301 | 1.56E-158 | 478 |
| OreG0009447.1 | Q8L838 | COG4 | 79.408 | 743 | 0 | 1231 |
| OreG0020451.1 | Q42656 | Alpha-galactosidase | 81.199 | 367 | 0 | 632 |
| OreG0013815.1 | Q8LPF3 | PME68 | 77.068 | 266 | 4.15E-153 | 436 |
| OreG0015919.1 | O04369 | RAC1 | 88.06 | 67 | 1.46E-35 | 121 |
| OreG0027239.1 | Q8VWI1 | ERD2B | 80.672 | 119 | 3.06E-66 | 203 |
| OreG0016430.1 | Q9FKS0 | UKL1 | 87.727 | 220 | 9.77E-135 | 391 |
| OreG0003240.1 | K7K424 | DGAT2D | 75.556 | 315 | 0 | 509 |
| OreG0026701.1 | Q9LS42 | CASP | 82.148 | 689 | 0 | 1142 |
| **OreG0005060.1*** | Q9S7R3 | LSH10 | 83.893 | 149 | 2.58E-90 | 264 |
| OreG0023535.4 | Q9SLN5 | MAP1A | 80.789 | 380 | 0 | 660 |
| OreG0010064.1 | P93033 | FUM1 | 89.676 | 494 | 0 | 907 |
| OreG0008279.2 | Q9ZU91 | At2g01630 | 78.043 | 460 | 0 | 756 |
| OreG0027226.4 | F4JUL9 | ARPC4 | 94.667 | 150 | 3.61E-101 | 290 |
| OreG0010693.1 | Q9SE95 | FIP2 | 79.026 | 267 | 5.54E-153 | 431 |
| OreG0005685.1 | Q9LDX3 | UTR4 | 79.598 | 348 | 0 | 552 |
| OreG0023977.1 | O48818 | EXPA4 | 88.095 | 210 | 1.75E-139 | 393 |
| OreG0009894.1 | Q944I4 | GLYK | 75.393 | 382 | 0 | 609 |
| OreG0003474.3 | Q8LPJ4 | ABCE2 | 93.223 | 605 | 0 | 1177 |
| OreG0006276.2 | O23627 | At1g29880 | 81.884 | 690 | 0 | 1183 |
| OreG0011919.1 | Q39659 | Glyoxysomal | 77.04 | 723 | 0 | 1159 |
| **OreG0019041.4*** | Q84XU2 | PAPP5 | 78.612 | 533 | 0 | 856 |
| OreG0003441.1 | Q9FJH0 | RABA1F | 92.593 | 216 | 2.20E-148 | 414 |
| OreG0020997.2 | Q9SX85 | SEC3A | 90.047 | 844 | 0 | 1557 |
| OreG0001557.1 | Q5S2C3 | PIR | 84.28 | 1285 | 0 | 2252 |
| OreG0018146.1 | Q9SEI3 | RPT4A | 93.467 | 398 | 0 | 761 |
| **OreG0020765.1-** | Q8S3N1 | SINAT5 | 81.818 | 297 | 0 | 518 |
| **OreG0019513.1-** | F4JY11 | CAND7 | 78.409 | 440 | 0 | 701 |
| OreG0020902.1 | Q9CAI3 | CAD1 | 76.353 | 351 | 0 | 562 |
| OreG0025586.3 | Q56YN3 | NADK1 | 75.098 | 510 | 0 | 775 |
| OreG0015243.1 | H2DH22 | Cytochrome | 78.652 | 534 | 0 | 858 |
| OreG0008794.1 | Q84WV1 | CCT3 | 83.633 | 556 | 0 | 920 |
| OreG0002311.2 | Q9SKZ5 | At2g32040 | 78.669 | 511 | 0 | 726 |
| OreG0000222.1 | Q9FJK7 | CYCC1-2 | 77.778 | 153 | 6.07E-86 | 256 |
| OreG0004781.1 | Q9LZD3 | EXO70A1 | 75.776 | 644 | 0 | 981 |
| OreG0025572.1 | O82802 | SIR1 | 82.2 | 691 | 0 | 1188 |
| **OreG0017846.1+*** | Q9FN03 | UVR8 | 81.448 | 442 | 0 | 743 |
| OreG0024546.1 | P49608 | Aconitate | 90.089 | 898 | 0 | 1706 |
| OreG0020604.2 | B0M3E8 | UGE1 | 88.421 | 285 | 0 | 538 |
| OreG0007587.2 | O81716 | PPC4-2 | 75.301 | 332 | 0 | 547 |
| OreG0021440.4 | Q9LFP0 | APT5 | 84.459 | 148 | 6.00E-86 | 252 |
| OreG0022806.2 | Q43082 | HEMC | 86.667 | 285 | 0 | 518 |
| OreG0010143.2 | Q9LZQ0 | At3g62400 | 84.375 | 64 | 2.05E-34 | 114 |
| OreG0014428.1 | Q94BV7 | NDB2 | 77.055 | 584 | 0 | 924 |
| OreG0015868.4 | O24362 | PAG1 | 95.402 | 174 | 1.30E-123 | 350 |
| OreG0017580.2 | Q9LET3 | RBL20 | 79.522 | 293 | 3.99E-159 | 447 |
| OreG0021872.1 | Q9SA78 | At1g30630 | 84.028 | 288 | 0 | 524 |
| **OreG0021601.3*** | Q8RWG1 | ABC1K1 | 83.307 | 635 | 0 | 1071 |
| OreG0011711.2 | Q8LPJ5 | At5g14590 | 86.111 | 396 | 0 | 735 |
| OreG0021689.1 | P51568 | AFC3 | 77.273 | 286 | 3.05E-166 | 469 |
| OreG0006047.2 | Q9SKZ1 | PURA1 | 83.456 | 272 | 2.70E-163 | 458 |
| OreG0026733.2 | Q94C12 | At3g18410 | 83.962 | 106 | 6.72E-64 | 191 |
| OreG0002704.2 | O04151 | CRT1 | 84.68 | 359 | 0 | 615 |
| OreG0017375.1 | O50064 | FIM2 | 78.378 | 666 | 0 | 1069 |
| **OreG0008237.1+*** | Q9C9W9 | ADO3 | 79.556 | 631 | 0 | 1021 |
| OreG0013157.1 | O49884 | RPL30 | 86.607 | 112 | 1.05E-69 | 206 |
| OreG0004984.1 | Q9ZNT0 | SKD1 | 87.385 | 436 | 0 | 777 |
| OreG0011733.2 | O23657 | RABC1 | 85 | 200 | 9.42E-127 | 358 |
| OreG0001752.2 | Q0WQ41 | IP5P7 | 77.852 | 596 | 0 | 925 |
| OreG0021078.1 | Q8H0S9 | MPA1 | 83.221 | 888 | 0 | 1545 |
| OreG0026682.2 | O24617 | MSH2 | 76.539 | 942 | 0 | 1521 |
| OreG0026247.1 | O49605 | CYP21-1 | 79.464 | 224 | 4.57E-120 | 343 |
| OreG0013203.1 | Q9SVA6 | DRG3 | 92.141 | 369 | 0 | 692 |
| OreG0019036.1 | O49354 | COQ3 | 78.333 | 240 | 1.76E-146 | 418 |
| OreG0007558.2 | Q38942 | RAE1 | 81.375 | 349 | 0 | 597 |
| OreG0001610.1 | Q9SSD2 | PRP8A | 94.075 | 2363 | 0 | 4617 |
| **OreG0021653.2-** | Q94B65 | UTR7 | 81.115 | 323 | 0 | 528 |
| **OreG0020929.2-** | F4IDS7 | VPS18 | 81.781 | 988 | 0 | 1726 |
| OreG0007247.5 | Q52JK6 | VIP2 | 81.593 | 565 | 0 | 836 |
| OreG0007920.1 | O81122 | ETR1 | 89.31 | 739 | 0 | 1375 |
| OreG0002986.1 | Q9FWT2 | UBC16 | 90.683 | 161 | 1.37E-102 | 293 |
| OreG0020547.1 | Q9FJR9 | NMAT2 | 76.644 | 745 | 0 | 1156 |
| **OreG0003479.1-** | Q8VXZ5 | XEG113 | 75.781 | 640 | 0 | 1014 |
| OreG0016211.2 | Q84J75 | RGTB1 | 80.831 | 313 | 0 | 525 |
| OreG0026633.4 | Q66GQ5 | RWA3 | 82.963 | 540 | 0 | 944 |
| OreG0019394.2 | F4HXZ1 | BRO1 | 81.346 | 654 | 0 | 1050 |
| OreG0022839.1 | Q9SFU6 | CALS9 | 78.354 | 1908 | 0 | 3093 |
| OreG0001813.2 | A2ZVI7 | CPK1 | 81.974 | 466 | 0 | 826 |
| **OreG0003292.3*** | Q9ZNU6 | DET1 | 75.328 | 458 | 0 | 719 |
| OreG0015242.3 | Q9ZVW2 | HEN2 | 80.156 | 1023 | 0 | 1670 |
| OreG0010236.4 | Q8GZQ3 | PNP1 | 80.895 | 581 | 0 | 938 |
| OreG0009326.4 | P21616 | Pyrophosphate-energized | 87.845 | 543 | 0 | 970 |
| OreG0004770.2 | Q6DW76 | DGD1 | 85.128 | 585 | 0 | 1056 |
| OreG0027430.1 | Q9FH13 | At5g56260 | 78.313 | 166 | 1.41E-95 | 276 |
| OreG0001015.1 | P93841 | ISPE | 78.212 | 358 | 0 | 593 |
| OreG0027486.1 | Q680K8 | At1g55760 | 76.364 | 330 | 0 | 535 |
| OreG0022649.1 | Q40467 | Eukaryotic | 95.642 | 413 | 0 | 822 |
| **OreG0000784.1-** | P43287 | PIP2-2 | 83.624 | 287 | 3.44E-180 | 500 |
| OreG0006610.1 | Q9SDZ9 | PAP2 | 75.484 | 465 | 0 | 744 |
| OreG0001800.2 | Q9SMC2 | Acetolactate | 81.818 | 374 | 0 | 614 |
| OreG0001166.1 | P82163 | RPS13 | 76.923 | 169 | 3.27E-85 | 250 |
| OreG0001108.3 | Q93ZH0 | LYM1 | 75.796 | 314 | 3.14E-169 | 479 |
| OreG0004687.2 | Q8LD27 | PBA1 | 90.435 | 230 | 7.42E-159 | 442 |
| OreG0018531.2 | Q9ZS21 | GLXI | 85.405 | 185 | 3.47E-122 | 347 |
| OreG0022256.1 | O80501 | RABH1B | 93.269 | 208 | 7.42E-144 | 402 |
| OreG0002834.2 | P19446 | Malate | 88.855 | 332 | 0 | 601 |
| OreG0000721.1 | Q500U8 | TKPR1 | 78.438 | 320 | 0 | 533 |
| OreG0012200.1 | Q9FNQ1 | BRR2C | 81.414 | 2093 | 0 | 3552 |
| **OreG0021478.1+-** | O48946 | CESA1 | 87.175 | 1076 | 0 | 1927 |
| OreG0011595.2 | Q69V23 | CESA3 | 82.985 | 1099 | 0 | 1841 |
| **OreG0005249.1-** | Q9M146 | MGP4 | 78.09 | 356 | 0 | 556 |
| OreG0024396.7 | Q9FIU7 | BGLU41 | 77.181 | 298 | 1.68E-176 | 501 |
| OreG0010228.3 | F4KE63 | EMB2247 | 85.547 | 768 | 0 | 1402 |
| **OreG0015826.2*** | Q852L0 | HD16 | 76.975 | 595 | 0 | 978 |
| OreG0015671.4 | Q500W7 | PIGM | 75.915 | 328 | 0 | 523 |
| OreG0017778.1 | Q94AH3 | At1g71900 | 81.667 | 240 | 1.43E-142 | 406 |
| OreG0024496.1 | Q9LYA9 | CSP41A | 81.503 | 346 | 0 | 596 |
| OreG0016902.2 | Q94EI9 | At3g14410 | 79.822 | 337 | 0 | 535 |
| **OreG0002505.2-** | O22161 | FBX5 | 78.065 | 930 | 0 | 1410 |
| OreG0011266.1 | Q9XET4 | RPS7 | 82.199 | 191 | 3.32E-117 | 333 |
| OreG0015209.5 | Q9SUG3 | ITPK3 | 78.462 | 260 | 9.35E-134 | 387 |
| OreG0008163.2 | Q9ZPS7 | TMN3 | 91.649 | 467 | 0 | 872 |
| **OreG0015412.1+-** | Q9FKS8 | LHT1 | 77.931 | 435 | 0 | 728 |
| OreG0014008.3 | Q42971 | ENO1 | 88.972 | 399 | 0 | 732 |
| OreG0003847.1 | Q948Z4 | SN1 | 75.676 | 74 | 1.50E-35 | 118 |
| OreG0009396.3 | Q9FKV1 | ANTR5 | 75.501 | 449 | 0 | 682 |
| OreG0017531.1 | Q9M2S6 | SDIR1 | 75.362 | 276 | 4.41E-138 | 395 |
| OreG0013942.1 | Q9M427 | UBP1 | 83.836 | 365 | 0 | 645 |
| OreG0007708.2 | Q9FRL3 | At1g75220 | 81 | 400 | 0 | 658 |
| **OreG0019502.1-** | Q9ZUT3 | ALS3 | 82.51 | 263 | 1.24E-154 | 434 |
| OreG0003818.2 | Q9C6D2 | MTK | 78.771 | 358 | 0 | 610 |
| OreG0019769.3 | Q75IM9 | Os05g0125500 | 92.562 | 242 | 2.02E-166 | 469 |
| OreG0020476.1 | Q9FX21 | STT3B | 86.765 | 748 | 0 | 1315 |
| OreG0001257.5 | Q0J7U6 | TPR2 | 77.47 | 1012 | 0 | 1657 |
| OreG0005016.1 | Q84N64 | ARC5 | 77.238 | 782 | 0 | 1222 |
| OreG0027160.2 | Q8S950 | NACK1 | 82.402 | 966 | 0 | 1620 |
| OreG0021652.1 | Q9SB75 | CSLC5 | 81.151 | 695 | 0 | 1145 |
| OreG0011290.7 | Q42093 | ABCC2 | 78.828 | 1450 | 0 | 2330 |
| OreG0009799.1 | Q9ZPI6 | AIM1 | 76.19 | 714 | 0 | 1106 |
| OreG0025343.1 | P27774 | Phosphoribulokinase, | 85.897 | 390 | 0 | 691 |
| OreG0008495.4 | Q9C6B2 | NRAMP2 | 83.824 | 272 | 1.19E-162 | 465 |
| OreG0010487.2 | Q9FKK7 | XYLA | 84.486 | 477 | 0 | 856 |
| OreG0016703.1 | Q9SIZ2 | At2g40290 | 88.462 | 260 | 1.15E-167 | 470 |
| OreG0023930.1 | O04015 | Delta-1-pyrroline-5-carboxylate | 77.954 | 694 | 0 | 1076 |
| OreG0013483.4 | Q9C509 | DPL1 | 82.963 | 405 | 0 | 724 |
| OreG0013628.2 | Q27GI3 | NAT6 | 82.682 | 537 | 0 | 919 |
| OreG0004465.1 | Q9LQI9 | UBP1B | 84.04 | 401 | 0 | 686 |
| OreG0024222.1 | Q9FKM3 | At5g57480 | 80.08 | 497 | 0 | 788 |
| OreG0017886.1- | Q9LTB0 | EXO84B | 76.601 | 765 | 0 | 1090 |
| OreG0024998.1 | Q9M099 | SCPL24 | 75.435 | 460 | 0 | 732 |
| OreG0009022.2 | Q9ZV87 | At1g78690 | 75.263 | 190 | 4.39E-105 | 306 |
| OreG0013363.1 | P25856 | GAPA1 | 86.667 | 405 | 0 | 676 |
| OreG0022757.1 | Q03194 | PMA4 | 88.819 | 948 | 0 | 1702 |
| OreG0024559.1 | P92979 | 1-Apr | 75.115 | 434 | 0 | 664 |
| OreG0015226.1 | Q9C9W0 | ABCI17 | 75.732 | 239 | 1.35E-132 | 379 |
| OreG0008998.1 | Q07796 | SODCC | 89.474 | 152 | 3.42E-95 | 274 |
| **OreG0022062.3+** | Q9FK88 | INVE | 76.158 | 583 | 0 | 922 |
| OreG0027821.5 | Q94A40 | At1g62020 | 85 | 1220 | 0 | 2186 |
| OreG0011812.1 | O80856 | ARPC1A | 79.032 | 372 | 0 | 610 |
| OreG0000079.1 | F4IF36 | FGT1 | 78.477 | 1287 | 0 | 2069 |
| OreG0027392.5 | Q9C9Z1 | At3g08650 | 78.984 | 433 | 0 | 650 |
| OreG0013825.1 | Q8LPF8 | OFUT29 | 76.2 | 521 | 0 | 809 |
| OreG0003760.2 | Q402F4 | TOM1 | 77.778 | 216 | 3.07E-122 | 351 |
| OreG0019267.3 | Q9ZVF7 | ESMD1 | 84.892 | 417 | 0 | 764 |
| OreG0003960.3 | Q9LZD3 | EXO70A1 | 80.657 | 548 | 0 | 858 |
| OreG0026699.2 | Q84WV1 | CCT3 | 90.288 | 556 | 0 | 1021 |
| OreG0012971.1 | Q9M2Y3 | RLP44 | 76.494 | 251 | 3.46E-134 | 385 |
| OreG0013973.1 | Q8RWQ9 | At3g45310 | 76.62 | 355 | 0 | 596 |
| OreG0020553.2 | O04979 | Lon | 85.408 | 747 | 0 | 1300 |
| **OreG0016118.2+-** | Q9LF50 | MEX1 | 75.177 | 282 | 4.50E-146 | 419 |
| OreG0021498.1 | Q8H156 | RAN3 | 99.548 | 221 | 1.11E-167 | 463 |
| OreG0006273.1 | Q9M9G7 | At1g71790 | 84.375 | 256 | 1.16E-160 | 448 |
| OreG0005285.2 | X5JA13 | SEC10a | 83.273 | 825 | 0 | 1366 |
| OreG0000332.1 | O04619 | ADNT1 | 79.494 | 356 | 0 | 587 |
| OreG0009851.3 | Q84WJ9 | At5g19680 | 78.882 | 322 | 0 | 519 |
| OreG0002805.4 | O48651 | SQE1 | 75.888 | 535 | 0 | 823 |
| OreG0017031.1 | Q9SAK4 | ALDH5F1 | 77.652 | 528 | 0 | 833 |
| OreG0020177.2 | Q9FRL8 | DHAR2 | 78.378 | 185 | 1.36E-104 | 302 |
| OreG0019475.1 | Q9SF40 | RPL4A | 83.744 | 406 | 0 | 697 |
| OreG0008052.2 | Q8RWY6 | CLASP | 76.349 | 1446 | 0 | 2154 |
| OreG0016519.1 | Q9FH05 | SCPL42 | 78.222 | 450 | 0 | 766 |
| **OreG0025977.3+-** | Q9FEL7 | LAX2 | 89.88 | 415 | 0 | 765 |
| OreG0026717.1 | Q8RXA7 | SCD1 | 76.582 | 1217 | 0 | 1909 |
| OreG0009257.1 | Q9FJH6 | ABCF1 | 87.726 | 554 | 0 | 1037 |
| OreG0023145.2 | Q9LV11 | AHA11 | 92.345 | 823 | 0 | 1585 |
| OreG0009273.1 | Q9SJZ6 | MED18 | 83.125 | 160 | 7.75E-100 | 289 |
| OreG0019987.1 | P21343 | PFP-BETA | 77.372 | 548 | 0 | 856 |
| OreG0011000.1 | B3H7G2 | URM1-2 | 83.838 | 99 | 2.45E-57 | 174 |
| OreG0011592.2 | Q9FIH8 | SPP | 78.693 | 1178 | 0 | 1920 |
| OreG0015968.1 | Q9STN8 | SINAT4 | 78.886 | 341 | 0 | 553 |
| **OreG0015037.1-** | Q9FHI1 | MOB1A | 92.727 | 55 | 1.50E-33 | 116 |
| OreG0007363.1 | Q0WQF4 | VPS53 | 81.884 | 828 | 0 | 1357 |
| OreG0017795.1 | Q56Y85 | MAP2B | 84.198 | 424 | 0 | 746 |
| OreG0004123.1 | Q9SY09 | SMD1B | 93.258 | 89 | 3.91E-55 | 170 |
| OreG0015643.1 | P35135 | Ubiquitin-conjugating | 98.649 | 148 | 1.10E-107 | 305 |
| OreG0013624.1 | C7A2A0 | BALDH | 79.213 | 534 | 0 | 909 |
| OreG0005008.1 | Q6ESZ9 | SDC1 | 83.991 | 456 | 0 | 809 |
| OreG0016302.1 | Q940J9 | At1g04430 | 78.447 | 631 | 0 | 1030 |
| OreG0005111.1 | Q40546 | Pyruvate | 76.265 | 573 | 0 | 895 |
| **OreG0012832.1-** | Q9LYC1 | GID1B | 79.24 | 342 | 0 | 574 |
| OreG0003730.1 | Q6Z1D5 | ATG8C | 94.444 | 108 | 1.10E-69 | 207 |
| OreG0003296.1 | O80585 | MTHFR2 | 81.176 | 595 | 0 | 1051 |
| OreG0000235.1 | Q9SV68 | CEQORH | 76.596 | 329 | 0 | 511 |
| OreG0007230.2 | O22644 | RPL23A | 87.013 | 154 | 1.89E-81 | 239 |
| **OreG0017899.1-** | Q9C512 | MNS1 | 77.385 | 566 | 0 | 890 |
| OreG0027419.2 | Q9ZVI9 | PECT1 | 86.901 | 313 | 0 | 566 |
| OreG0003952.2 | Q9CAF2 | NUDT26 | 77.027 | 148 | 6.23E-83 | 246 |
| OreG0002594.3 | O81098 | NRPB5A | 79.902 | 204 | 7.36E-122 | 346 |
| OreG0007401.1 | Q9C8P0 | EMB3003 | 76.433 | 471 | 0 | 639 |
| OreG0003013.7 | Q9SD33 | At3g51130 | 84.375 | 352 | 0 | 637 |
| OreG0007250.2 | P92939 | ECA1 | 80.845 | 757 | 0 | 1228 |
| OreG0001221.4 | Q38970 | ACC1 | 81.983 | 2259 | 0 | 3868 |
| OreG0017461.1 | Q94AA4 | PFK3 | 79.57 | 465 | 0 | 765 |
| OreG0003759.4 | Q9ZS88 | DER2.2 | 88.506 | 174 | 1.55E-111 | 320 |
| OreG0001798.2 | F4IS56 | ILK1 | 78.661 | 478 | 0 | 791 |
| OreG0019704.2 | Q0JBP5 | LOGL6 | 90.441 | 136 | 4.42E-88 | 259 |
| OreG0001561.1 | Q9M8Y5 | LTL1 | 77.686 | 121 | 4.40E-58 | 186 |
| OreG0021812.1 | Q6AST1 | HOX32 | 82.635 | 835 | 0 | 1435 |
| OreG0001698.1 | O23320 | CML8 | 86.301 | 146 | 3.45E-90 | 261 |
| **OreG0021277.1-** | Q9SJX7 | RUS2 | 77.209 | 430 | 0 | 704 |
| OreG0002233.1 | Q9LHE9 | VIII-1 | 78.862 | 1178 | 0 | 1885 |
| OreG0001168.1 | P27783 | NIA1 | 87 | 900 | 0 | 1649 |
| OreG0016263.1 | A7KTC5 | PT1 | 79.813 | 535 | 0 | 897 |
| OreG0019106.1 | Q9LT68 | NHD1 | 79.623 | 584 | 0 | 867 |
| OreG0026637.1 | P31926 | SUCS | 82.222 | 45 | 2.20E-19 | 82.4 |
| OreG0019175.3 | Q8RY81 | OFUT13 | 76.238 | 303 | 3.31E-178 | 502 |
| OreG0022307.4 | P94078 | At3g26720 | 75.649 | 809 | 0 | 1291 |
| OreG0018575.1 | Q9M8L9 | AVT6E | 79.04 | 396 | 0 | 624 |
| OreG0017896.2 | Q8H1G3 | MTPC4 | 85.574 | 305 | 0 | 527 |
| OreG0005367.1 | Q8W4L3 | BSK3 | 76.701 | 485 | 0 | 765 |
| OreG0012224.1 | Q96558 | UGD1 | 90 | 480 | 0 | 907 |
| OreG0019863.1 | Q02909 | PPC16 | 91.107 | 967 | 0 | 1819 |
| OreG0022284.1 | P51075 | CHS | 85.185 | 108 | 6.97E-66 | 206 |
| OreG0012688.3 | Q9C6M2 | At1g25530 | 80.645 | 372 | 0 | 598 |
| OreG0015815.3 | Q4V398 | RRT1 | 77.346 | 309 | 7.53E-180 | 509 |
| OreG0021120.3 | Q8LBU2 | GPX8 | 79.268 | 164 | 2.99E-97 | 281 |
| **OreG0013405.1-** | Q5W7F2 | AGD3 | 78.297 | 834 | 0 | 1308 |
| **OreG0014795.1*** | Q94JQ6 | CESA6 | 79.288 | 1096 | 0 | 1780 |
| OreG0022142.3 | F4HQM3 | NCER1 | 80.157 | 509 | 0 | 855 |
| OreG0015905.2 | Q9ZV56 | CCT1 | 78.355 | 231 | 1.57E-125 | 362 |
| **OreG0011003.2-** | Q949M9 | GET3A | 85.333 | 300 | 0 | 524 |
| OreG0005234.1 | Q8W4I4 | POT6 | 75.316 | 790 | 0 | 1217 |
| OreG0010783.1 | Q8W117 | SMU1 | 88.235 | 510 | 0 | 964 |
| **OreG0025319.1-** | Q9FHI1 | MOB1A | 91.628 | 215 | 1.30E-148 | 414 |
| OreG0025488.1 | Q76MV0 | B34 | 100 | 136 | 7.44E-95 | 272 |
| OreG0017472.2 | Q9LIG0 | At3g21360 | 76.615 | 325 | 0 | 516 |
| **OreG0025439.2-** | F4KD71 | DUR3 | 76.883 | 571 | 0 | 880 |
| OreG0014324.3 | Q9LEV3 | CBSX3 | 82.524 | 206 | 2.47E-124 | 352 |
| OreG0014938.1 | Q8GWE6 | EER5 | 80.244 | 410 | 0 | 687 |
| OreG0026045.1 | Q38953 | At3g26560 | 86.175 | 962 | 0 | 1705 |
| OreG0013262.1 | P42734 | CAD9 | 78.492 | 358 | 0 | 583 |
| OreG0010233.1 | Q8VXX4 | RFC3 | 85.311 | 354 | 0 | 645 |
| OreG0005021.1 | Q94F62 | BAK1 | 84.288 | 611 | 0 | 981 |
| OreG0004459.1 | Q9LJH5 | GLO4 | 75.766 | 359 | 0 | 580 |
| OreG0001416.2 | P49690 | RPL23A | 100 | 125 | 3.97E-87 | 252 |
| OreG0021358.1 | Q6S7B0 | TAF5 | 75.632 | 673 | 0 | 1039 |
| OreG0012891.2 | F4JGZ1 | MED16 | 75.82 | 1220 | 0 | 1796 |
| OreG0002546.2 | P69834 | ISPD | 75.773 | 194 | 1.87E-106 | 311 |
| **OreG0021690.2-** | Q67XG0 | GLB3 | 81.884 | 138 | 1.66E-82 | 243 |
| OreG0018149.3 | Q9SJT1 | SAE2 | 80.777 | 515 | 0 | 887 |
| OreG0022494.2 | Q8RUF8 | NLP3 | 75.51 | 294 | 2.96E-142 | 407 |
| **OreG0011862.2*** | Q9FEE2 | TON2 | 92.697 | 356 | 0 | 700 |
| OreG0011593.2 | O22446 | HDA19 | 85.653 | 467 | 0 | 807 |
| OreG0022691.1 | B5X0N6 | KEA6 | 84.991 | 533 | 0 | 890 |
| OreG0005233.3 | O80739 | POT12 | 78.561 | 681 | 0 | 1082 |
| OreG0014537.1 | Q9LIN9 | PAGR | 85.331 | 559 | 0 | 945 |
| OreG0017687.1 | O22874 | EXPA8 | 79.842 | 253 | 4.32E-151 | 424 |
| OreG0012998.2 | Q9FN32 | At5g53940 | 81.373 | 102 | 5.69E-61 | 186 |
| OreG0026942.1 | Q8L799 | MIOX1 | 76.568 | 303 | 2.36E-172 | 483 |
| OreG0021926.1 | Q39434 | RAB2BV | 85.116 | 215 | 1.85E-131 | 371 |
| OreG0022687.1 | Q944K3 | HDA2 | 77.035 | 344 | 0 | 556 |
| OreG0015323.1 | F4JP48 | MSH4 | 78.03 | 792 | 0 | 1285 |
| OreG0027212.1 | Q5YLB5 | GYRA | 77.447 | 940 | 0 | 1436 |
| OreG0018871.1 | Q9LI61 | AVT6A | 75.435 | 460 | 0 | 702 |
| OreG0011600.2 | Q8GYX8 | ATJ10 | 84.375 | 288 | 3.06E-178 | 500 |
| OreG0006763.6 | Q9SZG0 | HHP4 | 75.115 | 217 | 1.32E-120 | 350 |
| OreG0027231.1 | O64937 | REFA1 | 96.421 | 447 | 0 | 895 |
| OreG0000564.2 | Q8GZC3 | FAD2 | 83.812 | 383 | 0 | 667 |
| OreG0026957.3 | Q8L8B8 | LOG3 | 90.476 | 189 | 4.95E-125 | 354 |
| OreG0019408.3 | Q94AS9 | CNGC4 | 77.743 | 638 | 0 | 1018 |
| OreG0007361.2 | Q94AI6 | SEC6 | 90.048 | 623 | 0 | 1145 |
| OreG0022840.1 | Q9LXT9 | CALS3 | 83.498 | 1818 | 0 | 3114 |
| OreG0027691.1 | Q9ZNS1 | RPS7 | 83.158 | 190 | 4.81E-117 | 332 |
| OreG0011041.1 | Q9LPV9 | LWD1 | 91.908 | 346 | 0 | 653 |
| OreG0016754.2 | A5JTQ3 | Xyl2 | 78.291 | 585 | 0 | 966 |
| OreG0020356.1 | Q7XT99 | Os04g0338000 | 78.363 | 342 | 0 | 557 |
| OreG0020969.1 | Q9SVN5 | At4g13780 | 77.472 | 799 | 0 | 1303 |
| OreG0006816.1 | O48651 | SQE1 | 75.81 | 525 | 0 | 833 |
| OreG0014171.1 | O65201 | ACX2 | 84.844 | 673 | 0 | 1231 |
| OreG0006155.1 | Q08062 | Malate | 86.667 | 330 | 0 | 583 |
| OreG0015887.1 | Q8GUN2 | HINT1 | 79.592 | 147 | 4.69E-85 | 248 |
| OreG0014926.3 | Q9ZUN3 | KEA4 | 84.369 | 531 | 0 | 859 |
| OreG0024877.1 | Q8LKS5 | LACS7 | 75.578 | 692 | 0 | 1127 |
| OreG0008238.1 | Q8LF21 | DRP1C | 89.902 | 614 | 0 | 1154 |
| OreG0021517.1 | O48780 | KCS11 | 82.574 | 505 | 0 | 887 |
| OreG0020275.1 | P57720 | EMB1144 | 76.147 | 436 | 0 | 676 |
| OreG0019365.1 | P54967 | BIO2 | 80.526 | 380 | 0 | 634 |
| OreG0011759.1 | Q0WQE7 | 3-Aug | 80.154 | 519 | 0 | 864 |
| OreG0027234.1 | Q8W486 | OFUT1 | 76.426 | 526 | 0 | 834 |
| OreG0006617.1 | Q43636 | Thioredoxin | 77.391 | 115 | 2.98E-64 | 193 |
| OreG0008384.1 | Q8VYD8 | ORLIKE | 82.4 | 250 | 5.43E-152 | 428 |
| OreG0016143.2 | Q9ZSK1 | VTE4 | 76.471 | 289 | 4.93E-165 | 467 |
| OreG0023244.5 | Q9FI46 | CRL | 78.646 | 192 | 2.83E-116 | 333 |
| OreG0016405.3 | Q9FNA2 | PAO1 | 79.576 | 377 | 0 | 629 |
| OreG0024304.1 | P12333 | Chlorophyll | 89.888 | 267 | 1.37E-179 | 497 |
| OreG0025331.1 | P52581 | Isoflavone | 79.167 | 312 | 0 | 528 |
| OreG0023635.1 | P52780 | Glutamine--tRNA | 83.145 | 795 | 0 | 1374 |
| OreG0014201.1 | Q9LFU1 | ASN3 | 85.103 | 584 | 0 | 1057 |
| **OreG0019781.1-** | Q9FR44 | NMT1 | 83.673 | 490 | 0 | 875 |
| OreG0025783.1 | Q08069 | RPS8 | 89.593 | 221 | 1.90E-141 | 397 |
| OreG0017784.1 | Q8W3M6 | At4g06599 | 81.651 | 327 | 0 | 547 |
| OreG0002221.1 | P57742 | At5g23290 | 77.419 | 155 | 1.20E-80 | 238 |
| OreG0004974.1 | Q9SWB4 | PARP3 | 75.98 | 816 | 0 | 1309 |
| OreG0003059.3 | Q9FKW4 | CPK28 | 78.33 | 503 | 0 | 809 |
| OreG0020479.1 | Q8GTQ9 | SCOA | 85.119 | 336 | 0 | 574 |
| OreG0014185.2 | Q94FB9 | ABCD1 | 78.598 | 1341 | 0 | 2169 |
| OreG0005547.1 | Q9M3H5 | HMA1 | 78.966 | 580 | 0 | 938 |
| **OreG0011125.1*** | Q9FNP4 | PIA2 | 76 | 150 | 1.55E-80 | 239 |
| based on CNVs | |  |  |  |  |  |
| OreG0000118.9 | Q9CAT7 | At1g73230 | 79.464 | 112 | 3.06E-58 | 180 |
| OreG0009487.2 | O04350 | TFCA | 83.951 | 81 | 8.13E-45 | 143 |
| OreG0005021.1 | Q94F62 | BAK1 | 84.288 | 611 | 0 | 981 |
| OreG0025685.1 | Q9FMP5 | CPK17 | 82.927 | 533 | 0 | 924 |
| OreG0008350.1 | Q9SQZ1 | RPS17C | 78.571 | 140 | 7.03E-74 | 219 |
| OreG0019035.2 | P49299 | Citrate | 87.298 | 433 | 0 | 771 |
| **OreG0015037.1-** | Q9FHI1 | MOB1A | 92.727 | 55 | 1.50E-33 | 116 |
| OreG0022307.4 | P94078 | At3g26720 | 75.649 | 809 | 0 | 1291 |
| OreG0004643.1 | Q9XFK7 | MFT | 84.393 | 173 | 7.67E-108 | 308 |
| OreG0001845.1 | P51819 | HSP83A | 89.66 | 706 | 0 | 1203 |
| OreG0017087.1 | Q96282 | CLC-C | 79.352 | 586 | 0 | 937 |
| OreG0015496.3 | M1CZC0 | EBP1 | 87.267 | 322 | 0 | 559 |
| OreG0000908.1 | A0A2R6S148 | MYB1 | 80 | 80 | 1.66E-43 | 143 |
| OreG0003957.1 | Q9STT5 | ABCA7 | 77.778 | 126 | 2.44E-64 | 213 |
| OreG0026568.1 | P0CZ23 | ACX3 | 77.727 | 220 | 4.10E-129 | 382 |
| OreG0018619.1 | Q9LM02 | SMT1 | 75.781 | 128 | 2.18E-67 | 209 |
| OreG0025435.1 | P27164 | CAM53 | 83.938 | 193 | 1.74E-110 | 316 |
| OreG0015034.1 | O04011 | ABP20 | 79.327 | 208 | 1.31E-116 | 334 |
| OreG0023921.2 | Q9LZM7 | PRA1A1 | 76.51 | 149 | 5.54E-68 | 207 |
| OreG0014902.2 | Q6RJS2 | DHS | 81.614 | 223 | 9.95E-140 | 400 |
| OreG0015280.2 | O04006 | PSAH | 81.395 | 86 | 3.02E-44 | 143 |
| OreG0000009.1 | Q9FZL4 | MGD | 77.966 | 531 | 0 | 817 |
| OreG0020124.3 | Q9ZV36 | UXS6 | 89.565 | 345 | 0 | 631 |
| OreG0002906.1 | Q9ZRF1 | CAD | 86.402 | 353 | 0 | 613 |
| OreG0002766.1 | O82491 | SPT16 | 75.97 | 1057 | 0 | 1618 |
| OreG0001844.2 | Q9SW09 | RPS10A | 81.818 | 154 | 1.94E-78 | 234 |
| OreG0011039.3 | B9DGD6 | ACS | 81.369 | 628 | 0 | 1087 |
| **OreG0027649.1*** | Q75KD7 | AOC | 75.141 | 177 | 7.40E-96 | 280 |
| OreG0001808.1 | P13240 | PI206 | 75.843 | 178 | 1.76E-97 | 283 |
| OreG0002400.1 | O65554 | CIPK6 | 76.289 | 97 | 1.34E-35 | 128 |
| OreG0015536.1 | A1XJK0 | TIM22-4 | 76.596 | 141 | 3.09E-68 | 206 |
| **OreG0025831.2+** | Q5FB34 | ANR | 84.81 | 237 | 3.49E-153 | 432 |
| OreG0027604.3 | Q9AYE4 | LST8 | 86.709 | 316 | 0 | 589 |
| OreG0000785.3 | Q9ZRF1 | CAD | 75.284 | 352 | 0 | 534 |
| OreG0016711.1 | Q948P5 | Ferritin-4, | 83.099 | 213 | 4.40E-133 | 379 |
| OreG0010011.1 | Q9M9W1 | RPL22B | 84 | 125 | 2.07E-63 | 192 |
| OreG0026049.3 | Q6ZL17 | CSTLP2 | 88.079 | 302 | 0 | 543 |
| OreG0011440.1 | P25766 | RGP1 | 80.405 | 148 | 7.04E-87 | 256 |
| OreG0011320.1 | P54770 | TYDC3 | 76.667 | 90 | 8.01E-46 | 157 |
| OreG0000877.1 | O82616 | At4g10260 | 80.564 | 319 | 0 | 539 |
| **OreG0007630.1+** | O04173 | TAF10 | 84.559 | 136 | 3.62E-71 | 212 |
| OreG0026375.1 | P29766 | RPL8 | 94.972 | 179 | 4.14E-123 | 350 |
| OreG0016399.1 | Q9STT5 | ABCA7 | 76.19 | 126 | 5.73E-63 | 209 |
| OreG0019036.1 | O49354 | COQ3 | 78.333 | 240 | 1.76E-146 | 418 |
| OreG0022724.3 | O82197 | VPS32.1 | 77.13 | 223 | 5.98E-103 | 299 |
| OreG0006377.1 | P49317 | CAT3 | 80.752 | 452 | 0 | 785 |
| OreG0009081.1 | A8CDT3 | LUS | 82.09 | 67 | 3.29E-33 | 122 |
| OreG0009197.1 | Q9XIF2 | MTR4 | 76.22 | 164 | 1.74E-83 | 268 |
| OreG0018386.1 | Q5J907 | TCTP | 83.333 | 168 | 1.35E-103 | 296 |
| OreG0014901.1 | Q9M0E0 | RPS15AE | 82.946 | 129 | 8.81E-80 | 233 |
| OreG0000649.1 | Q76MV0 | B34 | 92.857 | 126 | 1.26E-78 | 231 |
| **OreG0001490.1-** | Q9LU77 | PIN2 | 75.556 | 90 | 4.44E-42 | 147 |
| OreG0003479.1- | Q8VXZ5 | XEG113 | 75.781 | 640 | 0 | 1014 |
| OreG0020637.5 | Q93ZQ5 | At4g22990 | 75.926 | 702 | 0 | 1077 |
| OreG0014765.1 | Q9SZ96 | URGT5 | 82.036 | 334 | 0 | 578 |
| OreG0001488.2 | Q9FFE0 | At5g16450 | 86.145 | 166 | 2.48E-104 | 298 |

**Table S17.** **Environmental variables used in this study.**

| **abbreviation** | **description** |
| --- | --- |
| BIO1 | Annual Mean Temperature |
| BIO2 (MDR) | Mean Diurnal Range (Mean of monthly (max temp - min temp)) |
| BIO3 | Isothermality (BIO2/BIO7) (×100) |
| BIO4 | Temperature Seasonality (standard deviation ×100) |
| BIO5 (MaT) | Max Temperature of Warmest Month |
| BIO6 (MiT) | Min Temperature of Coldest Month |
| BIO7 | Temperature Annual Range (BIO5-BIO6) |
| BIO8 | Mean Temperature of Wettest Quarter |
| BIO9 | Mean Temperature of Driest Quarter |
| BIO10 | Mean Temperature of Warmest Quarter |
| BIO11 | Mean Temperature of Coldest Quarter |
| BIO12 (AP) | Annual Precipitation |
| BIO13 | Precipitation of Wettest Month |
| BIO14 | Precipitation of Driest Month |
| BIO15 | Precipitation Seasonality (Coefficient of Variation) |
| BIO16 | Precipitation of Wettest Quarter |
| BIO17 | Precipitation of Driest Quarter |
| BIO18 | Precipitation of Warmest Quarter |
| BIO19 | Precipitation of Coldest Quarter |
| SR | Solar Radiation |
| SSF | Subsoil Sand Fraction |
| Sp | Subsoil pH |

**Table S18.** **The parameters of software used in this study.**

| **software** | **parameters** | **use for** |
| --- | --- | --- |
| fastp | -M 20 -5 -3 –l 50 | filter reads |
| BWA-MEM | default | map reads onto reference genome |
| GATK | described in the Methods section | SNP calling and hard filter |
| PHYLIP | default | construct neighbor-joining tree |
| IQ-TREE | -bb 1000 -m MFP -redo | construct gene tree |
| ASTRAL | default | merge gene trees |
| EIGENSOFT | -m 0 -t 0 | Principal Component Analysis |
| ADMIXTURE | default | generate population structure |
| BEAGLE | default | phase |
|  | ibd=true impute=false window=10000 overlap=1000 ibdtrim=100 | perform the Identity by Descent |
| msmc2 | default | investigate the demographic history using MSMC model |
| ANGSD | -only_proper_pairs 1 -uniqueOnly 1 -remove_bads 1 -minQ 20 -minMapQ 30 -doFasta 2 -basesPerline 100 -doCounts 1 | generate consensus sequences |
|  | -gl 1 -dosaf 1 -only_proper_pairs 1 -uniqueOnly 1 -remove_bads 1 -C 50 -minMapQ 30 -minQ 20 | for creating site frequency spectrum |
| fastsimcoal2 | -n100000 -M 1e-5 -w 1e-5 -l 10 -L 40 --msfs | investigate the demographic history of different models |
| vcftools | defalut | calculate FST and π |
| FastEPRR | defalut | calculate ρ |
| Control-FREEC | coefficientOfVariation=0.062;degree=3;minExpectedGC=0.35;maxExpectedGC=0.55;ploidy=2;telocentromeric=0 | identify Copy Number Variation region |
| SnpEff | default | annotate SNP |
| MAXENT | described in the Methods section | Predict the potential distribution of species |
